# Unravelling habituation for COVID-19-related information: A panel data study in Japan

**DOI:** 10.1371/journal.pone.0306456

**Published:** 2024-07-30

**Authors:** Shinya Fukui

**Affiliations:** Graduate School of Economics, Osaka Metropolitan University, Sakai, Osaka, Japan; National Institute of Informatics, JAPAN

## Abstract

This study examines people’s habituation to COVID-19-related information over almost three years. Using publicly available data from 47 Japanese prefectures, I analyse how human mobility responded to COVID-19-related information, such as the number of COVID-19-infected cases, the declaration of a state of emergency (DSE), and several doses of vaccine using an interactive effects model, which is a type of panel data regression. The results show that Japanese citizens were generally fearful and cautious during the first wave of the unknown infection. As such, a 1% week-on-week increase in the number of infected cases results in a decrease in human mobility by 1.09-percentage-point (pp) week-on-week. However, they gradually became habituated to similar infection information during the subsequent waves, which is reflected in 0.71 pp and 0.29 pp decreases in human mobility in the second and third waves. Nevertheless, the level of habituation decreased in response to the different types of the infection, such as new variants in the fourth wave, with 0.50 pp decrease. By contrast, regarding the DSE, it is more plausible to consider that human mobility responds to varying requests rather than habituate them. Whereas a rapid vaccination program could alleviate people’s concerns. I also find spatial spillovers of infection information on human mobility using a spatial weight matrix included in the regression model. However, there is no evidence of DSE or vaccination spatial spillovers, likely because both are valid only in one’s own prefecture. The implementation of flexible human mobility control policies by closely monitoring human mobility can prevent excessive or insufficient mobility control requests. Such a flexible policy can efficiently suppress infection spread and prevent economic activity reduction more than necessary. These implications are useful for evidence-based policymaking during future pandemics.

## Introduction

The COVID-19 pandemic has had significant socio-economic impacts at the global level as well as in Japan [1, 2]. Public health interventions (PHIs), such as lockdowns, have been employed to suppress COVID-19 infections by restricting people’s outgoing behaviour [3–10]. Additionally, according to [11–13], when infection spreads, people restrict their own outgoing behaviour, regardless of whether PHIs are issued, to reduce risks. For example, [12] show that, from March to May 2020, in the U.S., customer visits to the stores fell by 60%, 7% of which was explained by lockdowns, and the rest by own travel restrictions, owing to fear of infection.

As of June 2022, Japan experienced six waves of COVID-19 infection [14, 15], and the government has issued four declarations of a state of emergency (DSE) as a PHI [16] between 2020 and 2021. In 2021, the government rolled out a rapid vaccination programme [17, 18]. Additionally, the development of therapeutic agents has started worldwide [19], while much more has become known about post-infection effects [20]. However, new variants have emerged successively. Due to such a long-term pandemic, many individuals have grown tired of the COVID-19 pandemic, exhibiting the so-called ‘pandemic fatigue’ [21].

Throughout the COVID-19 pandemic, people have been exposed to two main pieces of information: the increase in the number of COVID-19 infections and PHIs, both leading them to feel fear and caution. As such, they changed their outgoing behaviours based on these two pieces of information [22–25]. Several studies thus explore the behavioural variations in response to COVID-19-related information over time. For example, [23, 26] show that Japanese citizens gradually reduced their stay-at-home response despite the repeated infection-increasing phase in 2020. Similarly, using data from 124 countries, [21] observes a gradual decline in adherence to social distancing under the continued PHIs from March to December 2020. Other studies [27, 28] find that the extent of curtailment of going-out behaviours slowly decreased from the first to the fourth DSE in Japan.

This study uses publicly available human mobility data and analyses how human behavioural responses to COVID-19-related information vary over the longer term. The significance of long-term analysis is twofold. First, I explore whether people became habituated to the COVID-19-related information. Previous studies have not adequately validated human mobility responses to multiple waves of infection. Second, I verify the response to new information, such as multiple vaccination doses or the emergence of new variants of COVID-19. The effect of vaccination on mobility has already been considered in the Appendix in S1 File of [28], which uses the number of vaccinations at least once per population. However, this study differs from [28] in that the vaccination is divided into first, second, and third doses, and the effects of each dose on human mobility are examined. Furthermore, the effects of new variants have also not been adequately investigated to date.

As such, human behavioural changes in response to COVID-19-related information over almost three years are still under-researched. One limitation is that the data are aggregated by prefecture and do not reveal the diversity of behaviours within a prefecture or for each individual. In addition, there is a lack of data on each variant’s spread status, infection speed, and severity for prefectures. Even considering these limitations, using panel data from 47 prefectures, analysing how people change their behaviour for repeated infection waves, new variants, repeated DSEs, and repeated doses of vaccine is crucial for evidence-based policymaking (EBPM) for future pandemics.

Habituation [29, 30] refers to the diminishing reaction to a repeated stimulus over time. As discussed in [31], in this study’s case, the stimulus is represented by fear and caution. For instance, a study [32] using individual questionnaires shows that COVID-19 anxiety became habituated over sixteen months. To investigate whether habituation has occurred, first, I examine the variation in human mobility in response to COVID-19 infection information over six waves; second, I re-examine the impact of multiple DSEs on human behaviour. As a PHI in Japan, a DSE does not legally regulate behaviour. Accordingly, the decision to restrain oneself from going out is voluntary (see Details of DSEs in Appendix A in S1 File).

Further, vaccination affects people’s fear and caution. As [33] show that vaccination influences future human behaviour, whether increased vaccination rates decrease people’s fear and caution of infection and promote outgoing behaviours is analysed. I also examine the spatial spillovers between prefectures, as changes in human mobility in one prefecture are influenced by information from other prefectures, using cross-prefecture travel (representing spatial interaction). Several studies [27, 34, 35] have noted the importance of considering regional spatial interaction when investigating the impact of COVID-19 infection or PHIs. As such, I incorporate a cross-term using a spatial weight matrix, which has not been used in previous related studies, to illustrate the spatial interactions between prefectures. Moreover, a single wave of infection comprises an increasing and decreasing phase. The difference between the two is that people are more likely to stay at home in the increasing phase but will gradually resume going out in the decreasing phase. Human mobility responses are considered heterogeneous across the two phases. I validate this conjecture for the first time by identifying the start, end, and peak of infection of different waves in each prefecture in Japan. I utilise the interactive effects model of Bai [36] in this study’s regression analysis, which is to control for the unobservable factors which affect human mobility.

Exploring how human mobility responded to (a) three pieces of information (i.e. repeated waves of infection or new variants, several DSEs, and several doses of vaccine), (b) the spatial spillovers of these three pieces of information, and (c) the different infection phases is important in considering mobility control policies over a long-term pandemic. While this study does not directly examine individual attitudes, it indirectly explores people’s habituation from COVID-19-related information, which arises due to decreased fear and caution, by assessing how human mobility responses changed during the pandemic.

## Materials and methods

### Data description

#### Human mobility

The daily human mobility data for each prefecture used in this study are obtained from Google’s COVID-19 Community Mobility Reports [37], which are composed of six human mobility categories: *retail & recreation*, *grocery & pharmacy*, *parks*, *transit stations*, *workplaces*, and *residential*. The data show the percentage change compared to a day-of-the-week baseline calculated based on median values for each day of the week for the five weeks from 3 January to 6 February 2020—just prior to the global outbreak of the COVID-19 pandemic.

**Fig 1:**
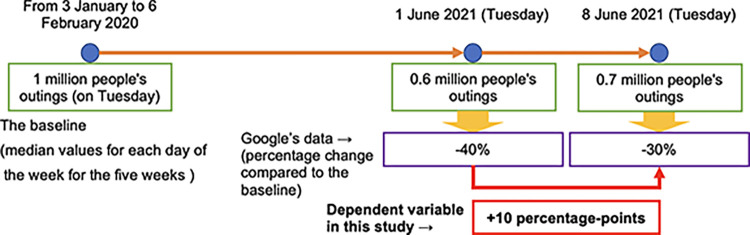
Example of a dependent variable. The author created fake data for the baseline, 1 June 2021 (the previous week), and 8 June 2021.

Since human mobility, the dependent variable in the estimation, has day-of-week fluctuations (i.e. each day of the week has its own variation characteristics, such as large variations during weekends), I take the difference from the previous week of the percentage change from the baseline. By taking the week-on-week difference, rather than using the percentage change from the baseline itself, one can capture the effect of new information in the short term, such as a week-on-week change in the number of daily newly infected cases, on people’s decisions about whether to go out. An example of a dependent variable is shown in Fig 1. Fig 1 depicts fictitious data on human mobility using Tuesday as an example, describing the situation on 8 June 2021 (on the right-hand side of the figure). The previous week was 1 June 2021 and the baseline is shown on the left-hand side of the figure. Published Google data are presented as percentages in purple boxes. My calculation is shown in the red box, which indicates a 10 percentage point (pp) change from the previous week.

#### Infected cases of COVID-19

Data on the daily number of newly infected cases of COVID-19 are obtained from NHK (*NIPPON HOSO KYOKAI*; Japan Broadcasting Corporation) [38]. Given that the number of new cases fluctuates over a vast range, it is better to take the logarithm of the data to mitigate heteroscedasticity. Since the data contain zeroes, following [4, 12, 22, 26], I use an inverse hyperbolic sine (IHS) transformation, which converts zeros to zeros and behaves similarly to a logarithm. Let *I*_*it*_ denote new cases; then, the IHS transformation of *I*_*it*_, $I_{it}^{*}$ becomes

$$I_{it}^{*}=\ln\left(I_{it}+\sqrt{I_{it}^{2}+1}\right).$$

Additionally, since new cases have day-of-week fluctuations (e.g. fewer PCR tests on weekends), I convert the IHS transformation of new infections to the difference from the same day of the previous week. The week-on-week difference of daily new infected cases transformed by the IHS, $\Delta I_{it}^{*}$, approximates the growth rate of new cases compared to the previous week.

According to an NHK news article [39], ‘A record number of cases—5,773—were confirmed in Tokyo on Friday, 13 August 2021. This is the highest number ever recorded. The number of cases has increased by 1,258 since last Friday, and the rapid spread of infection continues’ (translated from the Japanese article by the author). Daily news in Japan primarily covered the number of daily new infections and week-on-week changes in the number of infections. In this way, the approximation of the week-on-week growth rate of new cases in the data for the estimation reasonably illustrates the information received by people judging the severity of the situation based on week-on-week changes in the number of infections.

#### The declarations of a state of emergency

**Fig 2:**
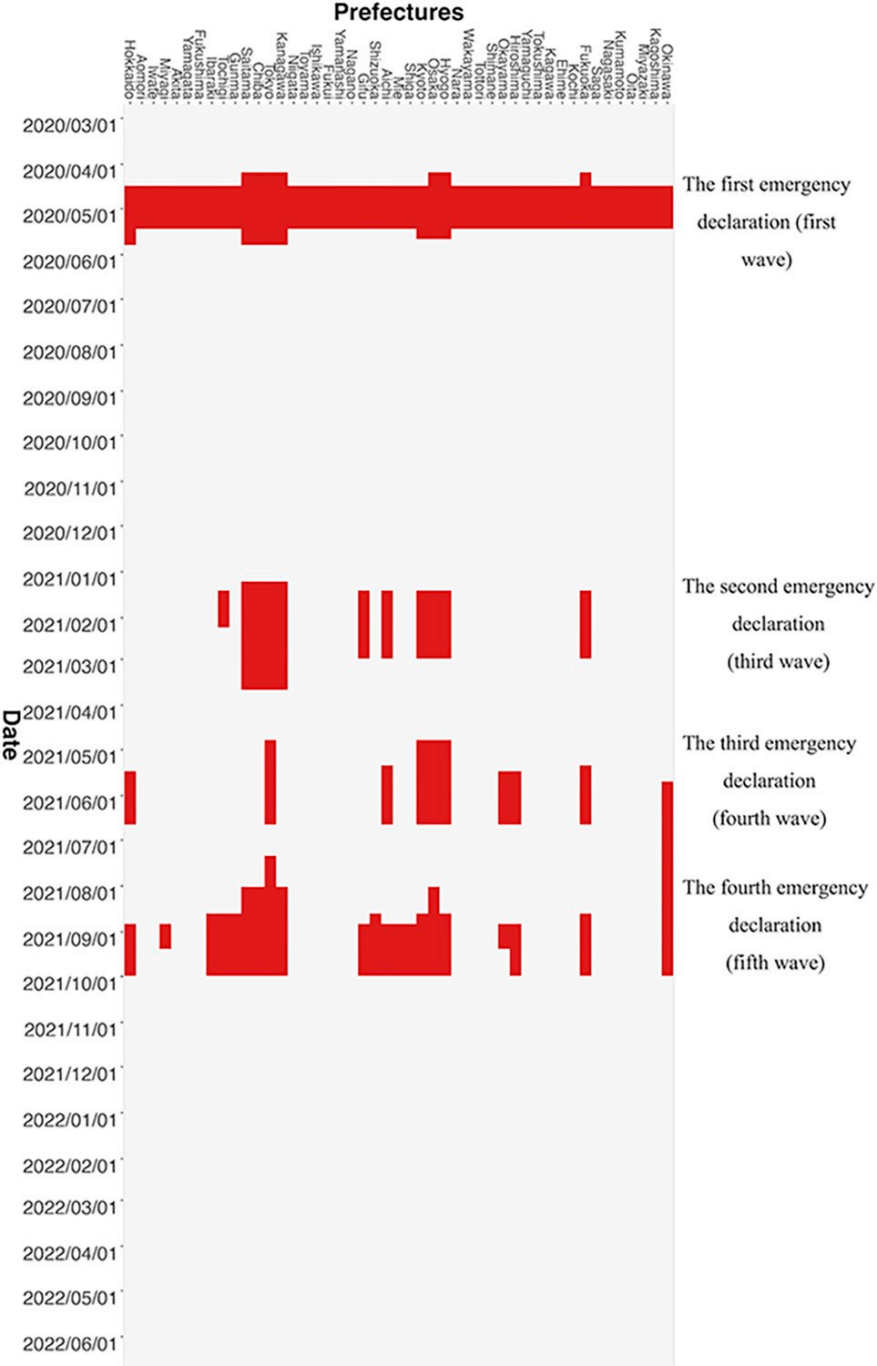
DSE periods for each prefecture. The red colour indicates the period during which the DSE was issued. The figure is constructed using the DSE data obtained from the Cabinet Secretariat’s COVID-19 Information and Resources [16].

The DSE data are obtained from the Cabinet Secretariat’s COVID-19 Information and Resources [16]. Fig 2 displays DSE periods for each prefecture in red. The timing of the DSE varies by prefecture, tending to be more frequent and longer in large urban areas such as Tokyo, Kanagawa, Saitama, Chiba, Osaka, Hyogo, Kyoto, and Fukuoka. The details of the DSEs are described in the Details of DSEs in Appendix A in S1 File.

#### Vaccination rates

The daily data on COVID-19 vaccination of each prefecture are obtained from the COVID-19 Vaccination Status by the Digital Agency [40]. I convert the data into a cumulative format to determine the vaccination rate per million persons. Population data for each prefecture (on 1 October 2020) are obtained from Population Estimates by the Statistics Bureau of Japan [41]. Since the number of vaccinations has day-of-week fluctuations, the data (vaccination rate per million persons) are converted to the week-on-week change. The other reasons for utilising week-on-week change are found in Reasons for utilising week-on-week vaccination rates in Appendix A in S1 File.

#### Control variables

The estimation model employs control variables. Specifically, I exploit temperature (average daily temperature) and precipitation (total daily precipitation) data from the Japan Meteorological Agency [42], which are relevant to human mobility. I select the capital of each prefecture as the geographical location for the daily temperature average and daily precipitation totals (there are only four missing data which I substitute with data from the nearest location). I use the IHS transformation for these two variables. Additionally, I take the difference from the previous day for the temperature.

Day-of-the-week dummies and weekends-and-holidays dummies (the latter is abbreviated as holidays-dummies) account for daily fixed effects. The day-of-the-week dummies are Tuesday, Wednesday, Thursday, and Friday. Holidays-dummies take a value of 1 if the day is Saturday or Sunday, a Japanese holiday, Lantan festival (O-bon in Japanese, which took place on 13–16 August for 2020, 2021, and 2022), or New Year’s holiday (from 29 December to 3 January, when most businesses and government offices are on vacation); otherwise, they take a value of 0.

As for prefectural fixed effects, two demographics—the population density per square kilometre of inhabitable land area and the percentage of the population over 65 years old—are extracted from the Regional Statistics Database (System of Social and Demographic Statistics) from the Statistics Bureau of Japan [43]. I take logarithms for these two demographics.

#### Human mobility and explanatory variables

**Fig 3:**
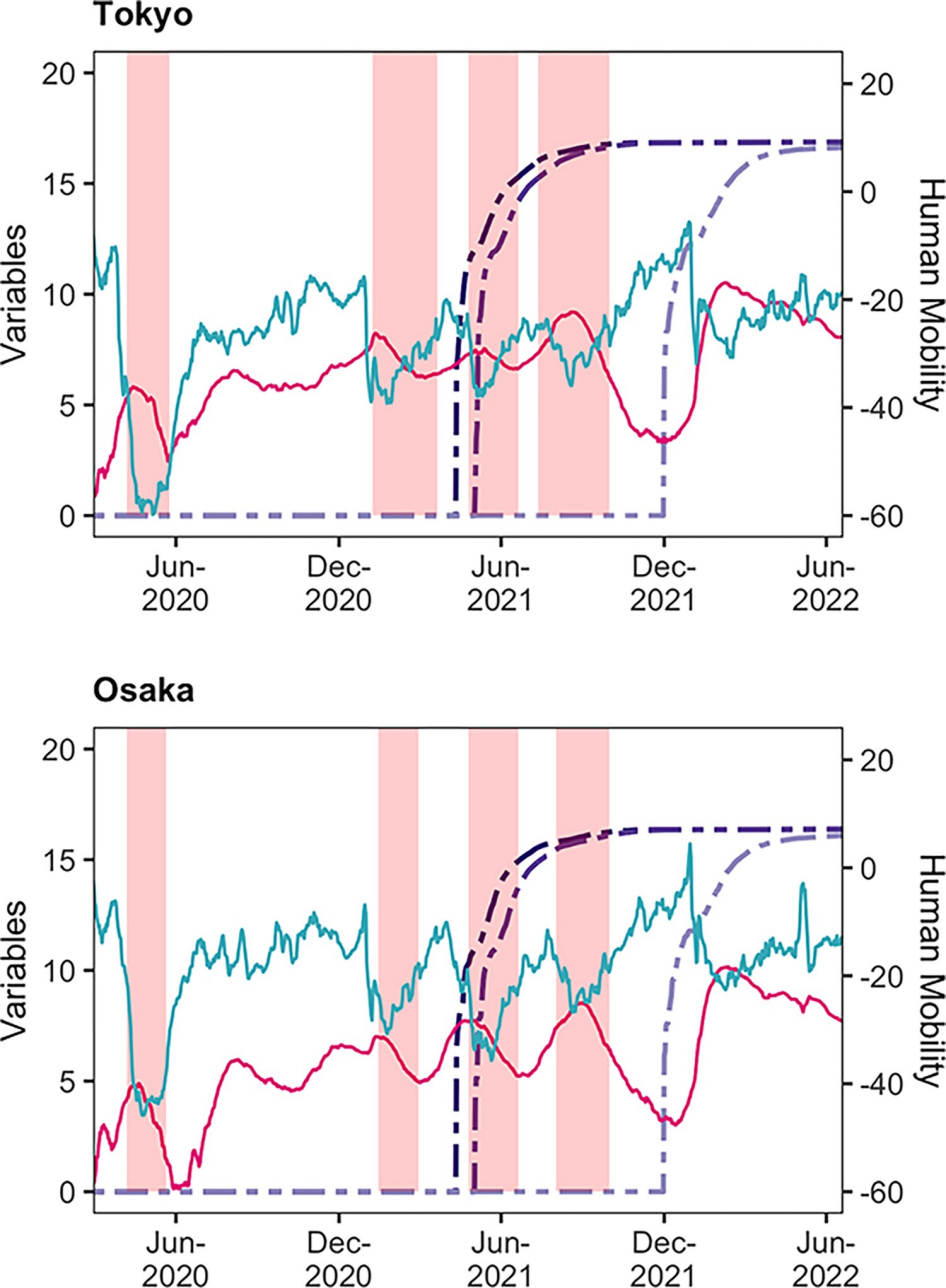
Time series plots of the variables for Tokyo and Osaka. On each chart, the blue-green-coloured line is the 7-day backward moving average using the geometric mean of human mobility in retail and recreation (on the right-hand axis); the red-purple-coloured line is the IHS transformation of the 7-day backward moving average number of infected persons (on the left-hand axis); the purple-coloured double-dashed lines are the IHS transformation of the cumulative number of people vaccinated with 1–3 doses (on the left-hand axis), and the pink-shaded areas are the DSE periods. The data transformation employed here, such as the 7-day backward moving average, is only for visualisation purposes; I use other transformations in the estimation. The figure is constructed using data from [16, 37, 38, 40].

Fig 3 displays time series plots of the variables for Tokyo and Osaka, two representative urban areas in Japan, from among the 47 prefectures (time series plots of the variables for all prefectures are in A1 Fig in Appendix A in S1 File). For human mobility, I use retail and recreation as a typical example. The figure shows an inverse relationship between the number of infections (red-purple-coloured line) and human mobility (blue-green-coloured line): as the number of infected people increases, human mobility tends to decrease, and vice versa. During the first infection wave, human mobility shows a sharp decline. Subsequently, when the DSEs were issued (pink-shaded areas), human mobility also declined sharply. In late 2021, human mobility recovered notably; however, human mobility decreased sharply, even without the DSE, when the number of infections surged around the beginning of 2022. The first and second vaccination doses (purple-coloured double-dashed lines) were administered rapidly, without any gap between them. The above trends are common between Tokyo and Osaka.

### Methodology

#### Regression model

This study uses Bai’s ‘interactive effects model’ [36]. Suppose that one has a panel dataset in which {(*Y*_*it*_, ***X***_*it*_)}, *i* = 1,2,⋯,*N*, *t* = 1,2,⋯,*T*; then, the interactive effects model is expressed as follows:

$$Y_{it}={X^{\prime }}_{it}\beta +v_{it},$$

$$v_{it}={{\lambda^{\prime }}_{i}F}_{t}+e_{it},$$ (1)

where *Y*_*it*_ is a dependent variable, ***X***_*it*_ is a *k*×1 vector of explanatory variables such that *k* is the number of explanatory variables, and ***β*** is a *k*×1 vector of the parameters to be estimated. ***λ***_*i*_ is a *d*×1 vector of factor loadings that differ for unit *i* and ***F***_*t*_ is a *d*×1 vector of a common factor that varies over time *t*, where *d* is the dimension of factors, and *e*_*it*_ is the error term. An unobservable term *v*_*it*_ has factor structure ***λ***′_*i*_***F***_*t*_ and random part *e*_*it*_.

Applying the interactive effects model (Eq (1)), the estimation model in this study becomes:

$${\Delta y}_{t}=\alpha +\sum_{s=1}^{6}\beta_{1s}{s\prime }_{st}\Delta I_{t-p}^{*increasing}+\sum_{s=1}^{6}\beta_{2s}{s\prime }_{st}\Delta I_{t-p}^{*decreasing}+\sum_{s=1}^{6}\gamma_{1s}{s\prime }_{st}W_{t-p}^{*}\Delta I_{t-p}^{*}+\sum_{s=1}^{6}\beta_{3s}{{s\prime }_{st}E}_{t}+\sum_{s=1}^{6}\gamma_{2s}{{s\prime }_{st}W}_{t}^{*}E_{t}+\sum_{v=1}^{3}\beta_{4v}{\Delta V}_{vt-p}+\sum_{v=1}^{3}\gamma_{3v}W_{t-p}^{*}{\Delta V}_{vt-p}+{\delta C}_{t}+\sum_{d=1}^{D}{\lambda_{d}F}_{td}+\phi W_{t}^{*}+\varepsilon_{t},$$ (2)

where Δ***y***_*t*_ is an *N*×1 (*N* = 47 prefectures) vector of the week-on-week difference in the percentage change from the baseline of human mobility or the residential time on day *t* (in pp). Variable ${\Delta I}_{t}^{*}$ is an *N*×1 vector of the week-on-week difference of new infections transformed by the IHS. This variable approximates the growth rate of new infections compared with the previous week. I separate ${\Delta I}_{t}^{*}$ for the increasing (${\Delta I}_{t}^{*increasing}$) and decreasing (${\Delta I}_{t}^{*decreasing}$) phases. ***s***_*st*_ is an *N*×1 vector of the step dummies, which takes 1 with each corresponding wave, *s* = 1,2,⋯,6. ***E***_*t*_ is an *N*×1 vector of the DSE dummy that takes the value of 1 under a DSE and 0 otherwise. Since DSEs were only issued for the first, third, fourth, and fifth waves, the DSE dummies for the second and sixth waves are zero. Variable Δ***V***_*vt*_ is an *N*×1 vector of the week-on-week change in the vaccination rate per million persons. Index *v* denotes the number of vaccine doses (from the first to the third dose) administered.

Here, *N*×*M* (where *j* = 1,2,⋯,*M*) standardised spatial weight matrix $W_{t}^{*}$ varies over time *t* (the standardised method is described below). $w_{ij}^{*}$ is an element of the standardised spatial weight matrix with row *i* (travel from) and column *j* (travel to), and diagonal element $w_{ii}^{*}$ is 0, and represent the weekly time-series changes in the movement of people between prefectures. Therefore, cross-terms $W_{t}^{*}\Delta I_{t}^{*},W_{t}^{*}E_{t}$, and $W_{t}^{*}{\Delta V}_{vt}$ exhibit the impact of information such as the number of infections, the DSE, and the vaccination rate from other prefectures, respectively. The larger the spatial weight matrix elements (more travel between prefectures), the larger the influence of information from other prefectures on one prefecture. None of the studies that have explored human mobility regarding COVID-19 have considered these spatial interactions.

Term ***C***_*t*_ is an *N*×*K* matrix of a control variable, where *K* is the number of control variables. *F*_*td*_ represents the common factors of dimension *d*; ***λ***_*d*_ is an *N*×1 vector where *d* = 1,2,⋯,*D* is the dimension of factors, representing factor loadings. This factor structure allows the proposed model to capture time-varying unobservable elements, such as people’s fear and caution of new variants emerging in some countries with different loadings over cross-sectional units. Meanwhile, ${\phi W}_{t}^{*}=\sum_{j=1}^{M}\phi_{j}w_{ijt}^{*}$ represents spatially weighted fixed effects, which was introduced by [44, 45]. Finally, ***ε***_*t*_ (*N*×1 vector) is an i.i.d. (independent and identically distributed) random component vector. ***λ***_*d*_, *F*_*td*_, and ***ε***_*t*_ are unobservable.

Parameters *α*, *β*_1*s*_, *β*_2*s*_, *β*_3*s*_, *β*_4*v*_, *γ*_1*s*_, *γ*_2*s*_, *γ*_3*v*_, ***δ*** (*K*×1 vector), and ***ϕ*** (*M*×1 vector) are to be estimated, while the parameters of interest are *β*_1*s*_, *β*_2*s*_, *β*_3*s*_, *β*_4*v*_, *γ*_1*s*_, *γ*_2*s*_, and *γ*_3*v*_ (***λ***_*d*_ and *F*_*td*_ are identified with some restrictions. See pp.1234-1238 in Bai [36] for more information).

#### Constructing a spatial weight matrix

To construct a spatial weight matrix, W*, I acquire a dataset called Cross-Prefecture Travel Data from Vital Signs of Economy-Regional Economy and Society Analyzing System (V-RESAS) provided by the Cabinet Secretariat and the Cabinet Office, Government of Japan [46] (note that V-RESAS is no longer available to the public [47]). These data are constructed from Agoop Corporation’s Current Population Data, which is based on GPS data obtained with user consent from specific smartphone applications and makes demographic data using day/night population data.

There are two types of data: *movement from other prefectures to the relevant prefecture* and *movement from the relevant prefecture to other prefectures*. In this study, I choose the former. Furthermore, there are two types of population movement: composition (%) and index. I choose the index because the index allows us to capture the decrease in movement compared to 2019 and the changes in the inter-prefecture movement for each prefecture. The index is based on the average movement across prefectures for all weeks in 2019 as 1. The ISO-8601 week number is employed in the index. According to V-RESAS, the index data is calculated as follows,

$$The\;index=\left(\frac{The\;population\;that\;moved\;from\;other\;prefectures\;to\;the\;prefecture\;during\;the\;week\;in\;question}{The\;average\;population\;that\;moved\;from\;other\;prefectures\;to\;the\;prefecture\;per\;week\;in\;2019}\right).$$

The spatial weight matrix ***W*** for the specific week is standardised using Kelejian and Prucha’s method [48]:

$$W^{*}=W\times \frac{1}{\min\left\{\underset{i}{\max}\sum_{j}^{M}w_{ij},\underset{j}{\max}\sum_{i}^{N}w_{ij}\right\}},$$ (3)

where *w*_*ij*_ is an element of the spatial weight matrix with row *i* (travel from) and column *j* (travel to), and the diagonal element *w*_*ii*_ is 0. This standardisation is conducted for all sample weeks. During the pandemic, the more people travel from prefecture *i* to prefecture *j*, the more the COVID-19 trend in prefecture *j* is expected to affect human mobility inside *i* substantially. Two factors can explain this: (1) the higher the interaction of the people between *i* and *j*, the higher the risk of COVID-19 transmission across prefectural borders; and (2) for commuters *i* to *j*, the trends in prefecture *j* are of concern. The larger the element of the standardised spatial weight matrix, $w_{ij}^{*}$, the higher the number of people moving between prefectures *i* and *j*.

Using the constructed standardised spatial weight matrix, $W_{t}^{*}$, I create the cross-terms, spatially weighted infected cases $W_{t}^{*}\Delta I_{it}^{*}$, spatially weighted DSE $W_{t}^{*}\Delta E_{it}$, and spatially weighted vaccination $W_{t}^{*}\Delta V_{it}$.

Regarding the impact of PHIs on human mobility, Ilin et al. [8] investigate the spatial spillover effects of PHIs on neighbourhoods. The authors consider certain distances for measuring the effects but not spatial interactions using spatial weights. Another study [27] uses a spatial error panel model to assess the effects of DSE on mobility. In constructing spatial weights, they use a nearest neighbour dummy that captures whether the other prefecture is close or not to a specific prefecture. In addition, [27] does not contain the spatial structure for the explanatory variables, as I do. This study differs from the above studies in that I employ spatial weight matrices constructed by the data related to human travel across prefectures, thus accounting for spatial interactions. In addition, I use cross-terms of spatial weights and explanatory variables to investigate the spatial spillover effects regarding each variable.

**Fig 4:**
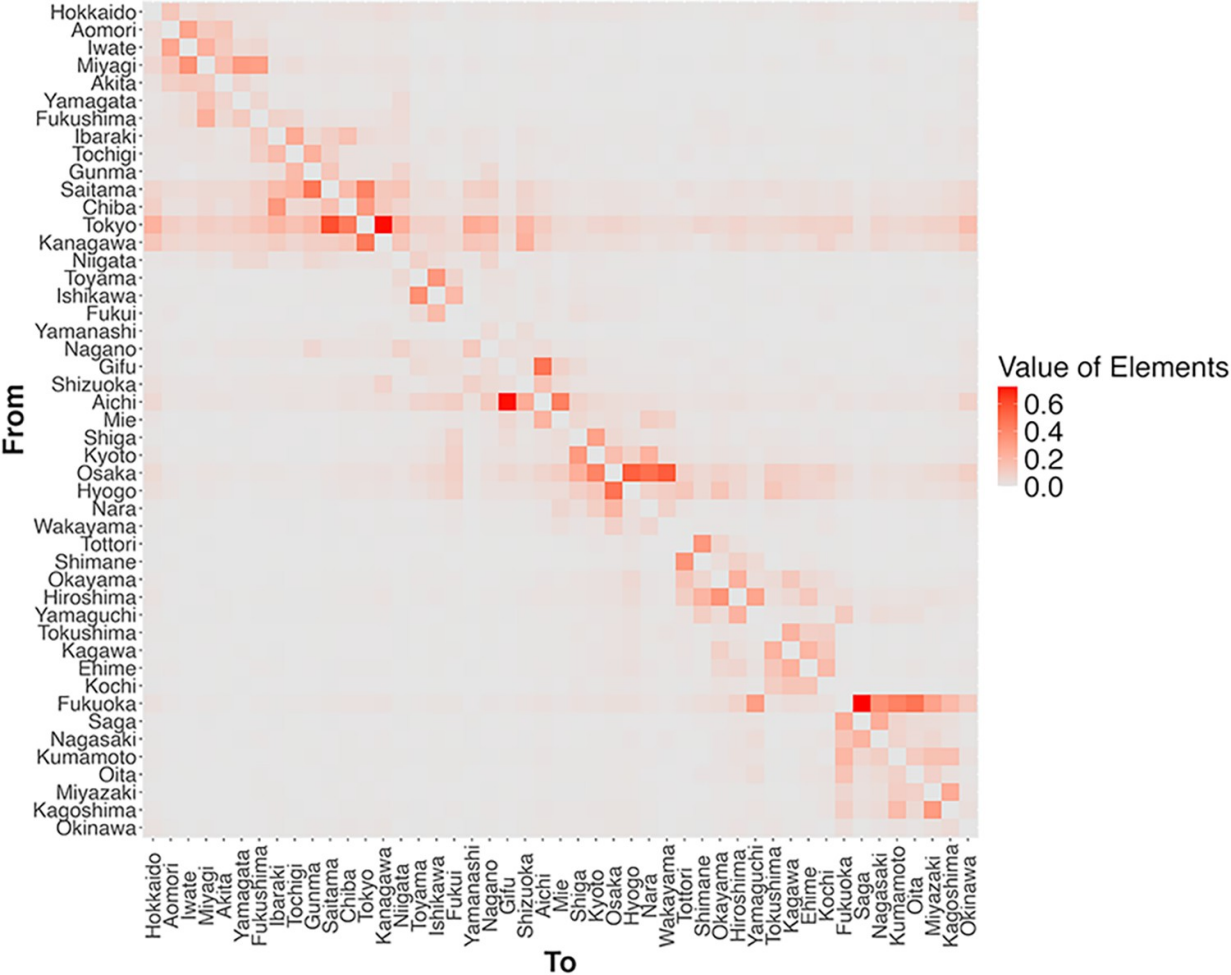
Spatial weight matrix per prefecture for the last week of January 2020 (27 January–2 February 2020). Within the figure, the darker the red colour, the greater the number of travellers between prefecture. Constructed using V-RESAS’s Cross-Prefecture Travel data from the Cabinet Secretariat and the Cabinet Office, Government of Japan [46] (note that V-RESAS is no longer available to the public [47]).

As an example, the elements of the spatial weight matrix for the last week of January 2020 (27 January–2 February 2020) are illustrated in Fig 4. This period occurred just prior to the pandemic when irregular movements due to the New Year celebrations in Japan had already dissipated; as a result, this week is representative of normal inter-prefecture travel. In Fig 4, the dark-red-coloured cells indicate that more people travel between prefectures located in or around large cities such as Tokyo, Aichi, Osaka, and Fukuoka.

#### Common factors and loadings

Common factors, denoted by *F*_*td*_, are unobservable elements that vary over time and are common for all cross-sectional units. Since each cross-sectional unit receives a different load from the common factor, ***λ***_*d*_ describes the difference in loadings. Component $\sum_{d=1}^{D}{\lambda_{d}F}_{td}$ corresponds to the generalisation of individual-specific and time-specific fixed effects in the panel data analyses; this component can better describe time-varying unobservable elements with different loadings through cross-sectional units than ordinary two-way fixed effects [36].

In fact, there are unobservable factors affecting human mobility that should be taken into account. For example, suppose a new variant of COVID-19 emerged in a country other than Japan. In that case, it is probable that people in urban areas such as Tokyo and Osaka, which have international hub airports and large population concentrations, would be more cautious of the outbreak than people in rural areas. Thus, when vigilant of the severity of a new variant infection, people in urban and rural areas will exercise different levels of caution in responding to such information.

Another example of an unobservable factor is how government policies are transmitted. For instance, even if there is an announcement by the Japanese government regarding vaccinations, each prefecture has a different system for promoting vaccinations, so residents in each prefecture (or, more specifically, each municipality, which is the main body promoting vaccinations) will receive the announcement differently. In addition, people may welcome the rapid vaccination programme announcement more in prefectures with higher infection rates.

Other examples that affect human mobility are starting working from home, or going back to work, which responds to the changes in the infection situation. Although these data are not available, the momentum for teleworking has likely been rising or subsiding across the prefectures and trends vary by prefecture. In general, the teleworking implementation rate is higher in large urban areas that include the three major metropolitan areas of Tokyo, Nagoya, and Osaka [49].

In such cases, these pieces of information are either unobservable or difficult to incorporate into the model. Additionally, such information affects all prefectures simultaneously; however, the level of sensitivity differs by prefecture. Therefore, the interactive effects model is a better method for controlling these unobservable factors. In the first example, common factors *F*_*td*_ capture the risk of epidemics of the new variants, while the loadings ***λ***_*d*_ capture differences between prefectures in vigilance against the new variants.

A Hausman-type specification test proposed by Bai [36] is used to determine whether it is appropriate to use the factors or classical two-way fixed effect; the results of all tests support the factor type. Dimension *d* of factors is chosen by consistent estimation for selecting the number of factors (Bai and Ng, [50]), which also considers the underestimation of the true variance of the penalty term. All estimations in this study show that dimension *d* = 7. For the variance-covariance matrix of the estimated parameters of the interactive effects model, I use heteroscedasticity- and autocorrelation-consistent estimators, proposed by Bai [36]. The estimation of the interactive effects model is conducted using the ‘phtt’ R library with circumstances of R 4.0.5.

#### Spatially weighted fixed effects

To control for the unobservable spatial spillovers resulting from the travel between each prefectural pair (other than spatially weighted infection, spatially weighted DSE, and spatially weighted vaccination, which are observable), I use the elements of the standardised spatial weight matrix W*, as if the least squares dummy variable (LSDV) estimation. In other words, $\sum_{j=1}^{M}\phi_{j}w_{ijt}^{*}$ represents spatially weighted fixed effects where cross-prefecture travel in each prefecture, $w_{ijt}^{*}$, varies over cross-sectional (prefectural) dimension *i* and time dimension *t* and *ϕ*_*j*_ is to be estimated. Unobservable spatial spillovers are related to human travel among prefectures, which are specific to the cross-sectional unit and vary over time.

#### Identifying the COVID-19 waves

As the government made no official announcements regarding the beginning and end of COVID-19 infection waves, I independently determine the COVID-19 wave duration of each prefecture.

The duration of the COVID-19 infection wave in each prefecture is determined using the 7-day backward moving average of new COVID-19 cases in each prefecture from 22 February 2020 to 15 August 2022 (using [38]). Due to data availability of Google’s mobility data, the first wave of infections began on 22 February 2020. The endpoint of each wave in each prefecture is the day with the lowest number of infections in that wave (in some cases, there may be several days in a row with the lowest number of infections; however, I assume that the subsequent wave begins the day after the first record low). In addition, I assume that the wave lasts from when the DSE is issued until it is lifted, even when a wave sees the lowest number of infections (the wave does not switch during the DSE). (The exception is Okinawa Prefecture, where DSEs corresponding to the third and fourth were issued without interruption.)

**Fig 5:**
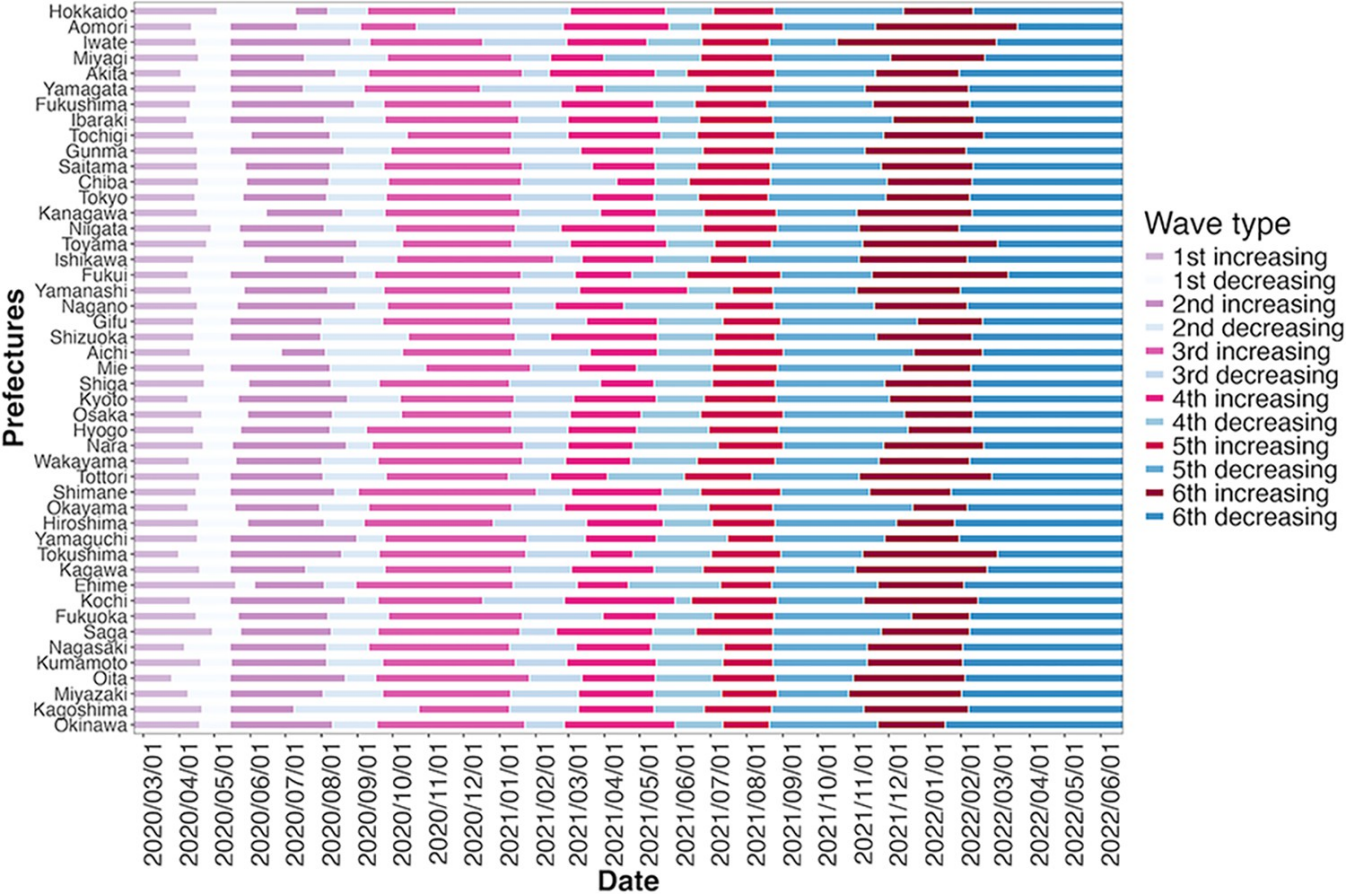
COVID-19 wave durations in each prefecture. The data are from 22 February 2020 to 20 June 2022. There are six waves in total, generated by taking a 7-day backward moving average of the number of daily new infections of COVID-19 from NHK [38].

In the first wave, in some cases, some prefectures repeatedly moved back and forth between having no infections and having infections. In such cases, it is difficult to determine the lowest infection; therefore, the wave’s endpoint is specified so that the wave does not deviate largely from those in other prefectures. In this study, it is necessary to identify the peak of each wave of infections to analyse each wave’s increasing and decreasing phases separately. The peak is defined as the day with the highest 7-day backward moving average of new cases in the wave (however, the peak could not be observed only for the first wave in Iwate Prefecture; accordingly, the average of the peaks of all prefectures’ waves serves as a proxy). As a result, as per Fig 5, six waves have been identified. The final point is specified as 20 June 2022, when the wave for all the prefectures (i.e. the entirety of Japan) hit a new low.

For clarity, the duration of the COVID-19 infection wave in each prefecture was determined as follows:

1. Using the 7-day backward moving average of new COVID-19 cases in each prefecture from 22 February 2020 to 15 August 2022.
2. The endpoint of each wave in each prefecture was the day with the lowest number of infections (assuming that the subsequent wave began the day after the first record low).
3. Assume that the wave lasts from when the DSE is issued until it is lifted (the wave does not switch during the DSE).
4. The peak is defined as the day with the highest 7-day backward moving average of the new cases in the wave.

#### Estimation lags

I take lags for the IHS difference (from the previous week) of new infections, $\Delta I_{t}^{*}$; its spatially weighted variables, $W_{t}^{*}\Delta I_{t}^{*}$; the week-on-week increased range of vaccination rate per million persons, Δ***V***_*vt*_; and its spatially weighted variables, $W_{t}^{*}{\Delta V}_{vt}$, in the estimation Eq (2). Daily lag *p* from day *t* is taken from 1 day (*p* = 1) to 7 days (*p* = 7). I also take a lag for the spatial weight matrix, $W_{t}^{*}$, which corresponds to lag day *t*−*p* of the estimation.

During the pandemic, most people decided whether to go out based on information regarding the infection status announced up to the previous day. The same is true for vaccination Δ*V*_*it*_, whereby outgoing behaviour is determined by the vaccines administered up to the previous day. Therefore, in this study, the maximum lag days is set to 7. By contrast, the lag is not taken for DSE (*E*_*it*_), as DSE on the day, rather than the day before or earlier, influences outgoing behaviour. Similarly, lags are not taken for the control variables: temperature, precipitation, day-of-the-week dummies, and holidays-dummies, since each is only relevant for human mobility of that day.

Another reason to take the lags for the infected cases is the announcement timing. Daily infected cases are only reported in the evening (around 17:00) or later. For instance, the infected case in Tokyo on 31 August 2021 was announced by NHK at 23:25, Nikkei at 17:00, Bloomberg at 17:19, Jiji Press News at 22:34, TV Asahi at 18:45, and Tokyo Shinbun at 16:56 (based on Google search which accessed on 15 August 2022). Therefore, people checked the infection information on the day and decided whether to go out starting from the following day onwards.

Due to the data used to create the weight matrix, the value is fixed for a given week. Therefore, in this study, I use a spatial weight matrix (W*) for the week corresponding to day *t* (or *t*−*p*) in the analysis, which gives $W_{t}^{*}$ (or $W_{t-p}^{*}$). For example, 15 June 2022 corresponds to the 24th week of 2022; then, the spatial weight matrix for the 24th week is selected. If one takes *p* = 1 lag, 14 June 2022 is obtained, which also corresponds to the 24th week of 2022. Therefore, one chooses the spatial weight matrix for the 24th week. Nevertheless, if lag *p* = 5 is taken from 15 June 2022, the observed date would be 10 June 2022, corresponding to the 23rd week of 2022, that is, the spatial weight matrix will be that of the 23rd week.

Estimates are conducted separately for each lag day: meaning that the estimation is performed seven times. A model encompassing all lag orders (distributed lag model) is also estimated to ensure robustness. I employ the polynomial degree 1 Almon lag model to avoid multicollinearity arising from the distributed lag model. Further details on the Almon lag model estimation are provided in Appendix B in S1 File.

#### Empirical approach

I use panel data from 47 prefectures over 850 days, from 22 February 2020 to 20 June 2022, for six infection waves. Lags (*p* in Eq (2)) are taken from 1 to 7 days for the week-on-week growth rate of new infections, week-on-week changes in vaccination rate, and the spatially weighted of these variables.

## Results

In this paper, I focus on the *retail & recreation* and *residential*. When avoiding unnecessary mobility, either in reducing the risk of infection or responding to the DSE by the government, people minimise their outings mainly to retail stores (not grocery stores and pharmacies, which are essential) and entertainment venues and also have a greater tendency to stay at home. In Google’s data, *residential* indicates time spent at home. Later, I extend the estimation and present the results for other mobilities in the Appendix D in S1 File. The empirical results for human mobility of retail and recreation are presented below.

### Retail and recreation human mobility response

**Fig 6:**
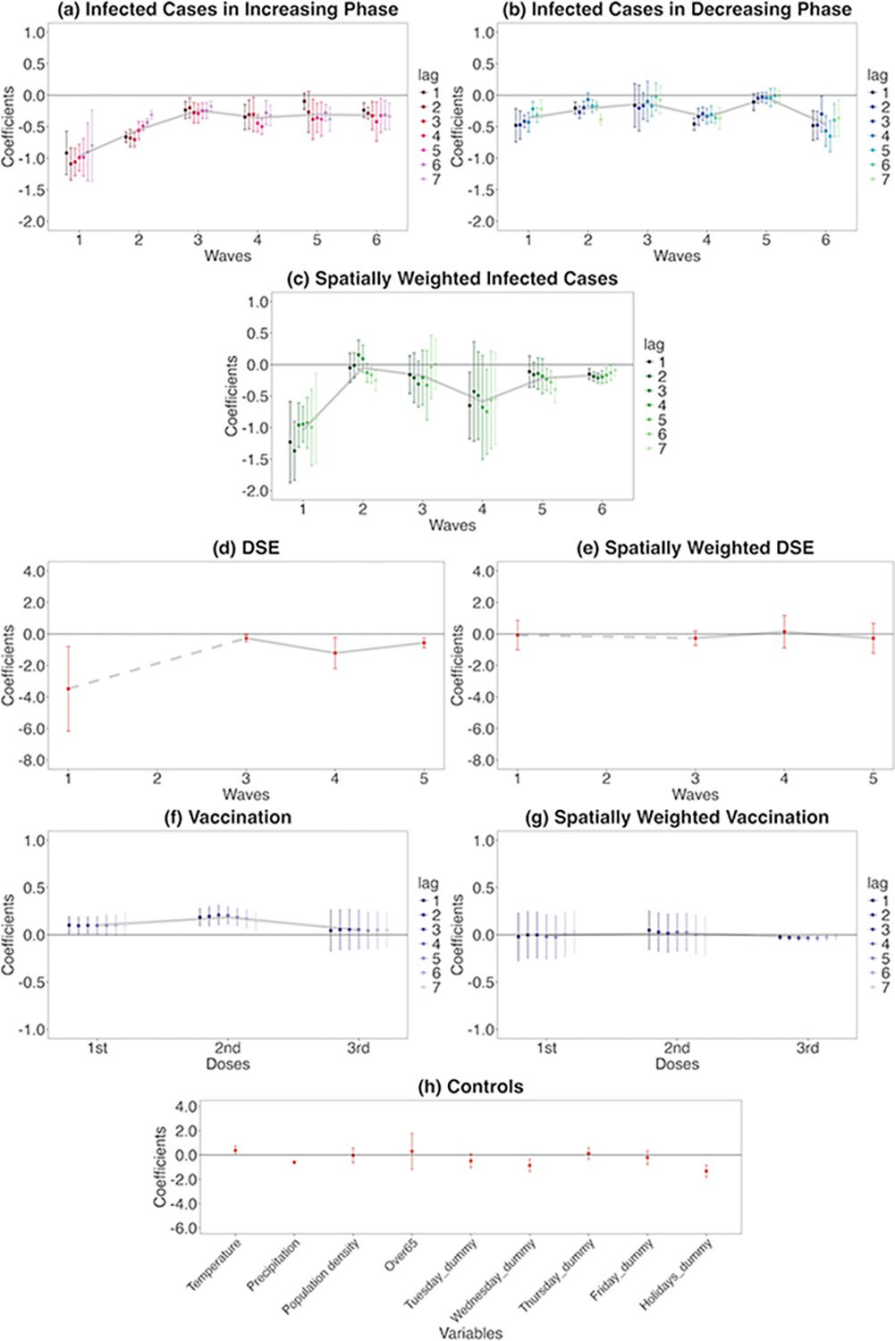
Retail and recreation human mobility responses to COVID-19-related information. On each chart, the points are estimated coefficients, and the bars indicate upper and lower 95% confidence intervals. The grey line traces the average coefficients of each lag day. There are six infection waves, but DSEs were only issued for the first, third, fourth, and fifth waves. I take a daily lag from 1 to 7 days for infected cases in the increasing phase, infected cases in the decreasing phase, spatially weighted infected cases, vaccination, and spatially weighted vaccination. I conduct the regression analysis seven times, from lags 1 to 7. I do not take a daily lag for the DSE, spatially weighted DSE, and control variables; these estimates are from the lag-1 regression.

Each graph in Fig 6 shows the weekly changes of the percentage change of human mobility from the baseline in response to each of the explanatory variables in Eq (2). In each graph, if the coefficient is positive, a point below zero on the vertical axis indicates a week-on-week decrease in the percentage change in human mobility from the baseline, and vice versa. The horizontal axis indicates each infection wave or number of vaccine doses. For the control variables, each variable is shown on the horizontal axis.

#### Response to infected cases in the increasing phase

As shown in Fig 6A, during the phase of increasing infections, a 1% week-on-week increase in the number of infected cases results in a decrease in human mobility by 1.09 pp week-on-week, at most (lag: 2, standard error [s.e.] = 0.13). The reduction in human mobility in the first wave weakened daily from 2- to 7-day lags. The 6- and 7-day lags have a wide confidence interval (CI) (95% CI for lag 7 = [-1.36 to -0.23]).

The extent of reductions during the second wave is smaller than that during the first, but it is still high with a maximum 0.71 pp decrease (lag: 3, s.e. = 0.06). The responses in the second wave also decreased daily, from 3- to 7-day lags. The third wave shows a more modest human mobility response than the first and second waves, with a maximum 0.29 pp decrease (lag: 4, s.e. = 0.08). The response remained flat for each lag day.

Meanwhile, the human mobility response rose in the fourth wave; the maximum decrease is 0.50 pp (lag: 5, s.e. = 0.06) and the reduction is strengthened on lag days 4 and 5. In the fifth wave, although lag days 1 and 2 do not meet the 5% significance threshold, human mobility is largely reduced from the 3- to the 7-day lags with a maximum 0.38 pp decrease (lag: 3, s.e. = 0.16). The maximum decrease in the sixth wave is 0.42 pp (lag: 4, s.e. = 0.16) and the response gradually intensified from lag day 1 to 4, remaining significant until lag day 7, with similar reductions as in waves four and five.

#### Response to infected cases in the decreasing phase

During the phase of decreasing infection (the recovery phase), as presented in Fig 6B, if the negative range in the estimated value is large, it indicates a prominent week-on-week increase in human mobility (the percentage change from the baseline), responding to the decreasing infected cases.

As a result, in the first wave, the negative range is relatively large. However, human mobility remains somewhat reduced, as the magnitudes of the estimates are smaller than those in the increasing phase. A 1% decrease in the number of infections will result in, at most, a 0.48 pp (lag: 1, s.e. = 0.13) increase in human mobility. In the second wave, the maximum is a 0.39 pp increase (lag: 7, s.e. = 0.04). Only lag 5 is significant at the 5% level for the third wave; however, the estimated coefficient is small.

By contrast, the decreasing phase of the fourth wave shows at most a 0.46 pp (lag: 1, s.e. = 0.05) increase in human mobility—a similar magnitude to that of the increasing phase (0.50 pp); it recovered to the same extent that it had reduced during the increasing phase during the fourth wave. However, in the fifth wave, the estimates are no longer significant and human mobility did not recover. Finally, the sixth wave shows the highest value of all waves, with a maximum increase of 0.65 pp (lag: 5, s.e. = 0.13).

#### Response to spatially weighted infected cases

The increasing and decreasing phases are not separated for the spatially weighted infected cases (Fig 6C). I find that individual going-out behaviours dramatically changed in response to infection information from the other prefectures in the first wave, with the maximum (in absolute value) being 1.37 pp (lag: 2, s.e. = 0.24). From the second to the third wave, the results are insignificant for most lags; the magnitudes are modest for those significant estimates. Conversely, in the fourth wave, lags 1 and 5 are significant at the 5% level; the maximum (in absolute value) is 0.75 pp (lag: 5, s.e. = 0.34). In the fifth wave, lags 5 to 7 are significant, with a maximum of 0.39 pp (in absolute value) (lag: 7, s.e. = 0.10). All lags are significant in the sixth wave, with the largest being 0.21 pp (in absolute value) (lag: 3, s.e. = 0.04).

#### Responses to the DSE and spatially weighted DSE

A DSE was only issued for the first, third, fourth, and fifth waves (Fig 6D). The first DSE (in the first wave) greatly reduced human mobility. Although the CI is relatively large, there is a 3.48 pp week-on-week decrease (s.e. = 1.37) in the percentage change from the baseline human mobility. Although significant in the second DSE (third wave), the magnitude is low (0.27 pp decrease, s.e. = 0.11). In the third DSE (fourth wave), the magnitude is large again, with a 1.21 pp decrease (s.e. = 0.51). The fourth DSE (fifth wave) lead to a slightly lower but still significant, with a 0.56 pp decrease (s.e. = 0.16). By contrast, the spatially weighted DSE is insignificant in any of the waves (Fig 6E).

#### Responses to vaccination and spatially weighted vaccination

Regarding vaccination, for a first vaccine dose, only some lags are significant, with each having a negligible impact (Fig 6F). The effect is more apparent for the second than the first dose, which is significant for all lag days. A 1 pp week-on-week increase in the vaccination rate leads to up to 0.21 pp week-on-week increase in the percentage change from the baseline human mobility (lag: 3, s.e. = 0.05). However, none of the results is significant for the third dose. Spatially weighted vaccination is not significant for any doses (Fig 6G, only lag 3 in the third dose is significant, while the magnitude is negligible).

#### Control variables

As for the control variables (Fig 6H), Wednesday and holiday dummies are significant. Additionally, the precipitation coefficient is negative and significant, meaning increased precipitation reduced human mobility.

### Residential time response

**Fig 7:**
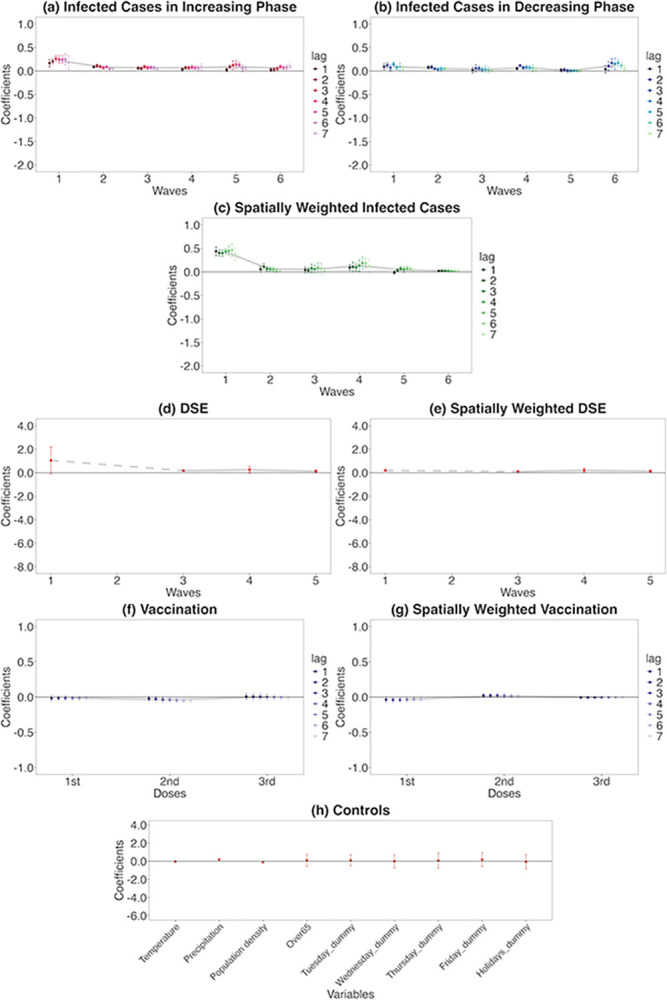
Residential time responses to COVID-19-related information. On each chart, the points are estimated coefficients, and the bars indicate upper and lower 95% confidence intervals. The grey line traces the average coefficients of each lag day. There are six infection waves, but DSEs were only issued for the first, third, fourth, and fifth waves. I take a daily lag from 1 to 7 days for infected cases in the increasing phase, infected cases in the decreasing phase, spatially weighted infected cases, vaccination, and spatially weighted vaccination. I conduct the regression analysis seven times, from lags 1 to 7. I do not take a daily lag for the DSE, spatially weighted DSE, and controls; these estimates are from the lag-1 regression.

The *residential* category indicates the change in the time spent at home. According to Google [51], given that people are already spending a large portion of their day at their residences, the change in time spent at home is not substantial (even on workdays) compared to the human mobility. As per Fig 7, the response is minimal; however, the trends are clear.

#### Response to infected cases in the increasing phase

A 1% week-on-week increase in the number of infected cases during the first wave is associated with at most a 0.26 pp (lag: 3, s.e. = 0.03) week-on-week increase in the percentage change of the residential time from the baseline (Fig 7A). In the second wave, the confidence interval is narrower, and the magnitude of the increase in the residential time is lower than in the first wave, with a maximum of 0.11 pp (lag: 2, s.e. = 0.02). Similar to the case of retail and recreation, the impact in the first and second waves decreased each day (from 3-day to 7-day lags in the first wave and from 2-day to 7-day lags in the second wave).

In the third and fourth waves, the effect is even more negligible, with a maximum increase of 0.09 pp in the third wave (lag: 3, s.e. = 0.02) and a maximum increase of 0.09 pp in the fourth wave (lag: 7, s.e. = 0.04). The fourth wave has a slightly increasing trend as the lag periods are longer. For all four waves, all of the results are significant at a 5% significance level. While for the fifth wave, the impact increase from lag 1 to lag 4, with a maximum increase of 0.14 pp (lag: 4, s.e. = 0.04). Similarly, for the sixth wave, an increasing trend is observed as the lag periods become longer, with a maximum increase of 0.10 pp (lag: 7, s.e. = 0.03).

#### Response to infected cases in the decreasing phase

In the decreasing phase (Fig 7B), when the estimates are positive and significant, means the residential time decreases as the infected cases drop. In the first wave, residential time is reduced by 0.14 pp at max (lag: 4, s.e. = 0.02). However, the impact is smaller than in the increasing phase. In the second wave, the impact is even smaller, with a maximum reduction of 0.08 pp (lag: 2, s.e. = 0.02). At the same time, the third wave is insignificant except for lag 3; furthermore, the impact of lag 3 is negligible. Conversely, in the fourth wave, the magnitude of the estimates increases slightly. Notably, the same tendency (response rose in the fourth wave) is observed in the retail and recreation case, with a maximum reduction of 0.12 pp (lag: 2, s.e. = 0.01). Again, the fifth wave is insignificant for all lag days, consistent with the retail and recreation case. The sixth wave, as in the case of retail and recreation, has a larger impact, with a 0.17 pp reduction (lag: 3, s.e. = 0.05).

#### Response to spatially weighted infected cases

For spatially weighted infected cases (Fig 7C), as for spatially weighted infected cases in retail and recreation, the impact of the first wave is large. The maximum impact is 0.46 pp (lag: 6, s.e. = 0.07) on residential time (i.e. residential time increases as the infected case increases in other prefectures and vice versa). In the second wave, the impact drops, with a maximum of 0.11 pp (lag: 2, s.e. = 0.03). In the third wave, all lags are insignificant at a 5% level. In the fourth wave, the estimates are slightly higher, with a maximum of 0.19 pp (lag: 5, s.e. = 0.07). Meanwhile, in the fifth wave, all lags are insignificant except lags 3, 5 and 6; these three lags all have negligible estimates. Finally, the sixth wave, while all lags are significant, the effect is relatively weak.

#### Responses to DSE and spatially weighted DSE

Although the estimates for the DSEs are positive, only the third wave is significant at a 5% level (estimated coefficient = 0.21, s.e. = 0.03) (Fig 7F). DSEs have relatively small impacts on residential time compared to retail and recreation. The spatially weighted DSE is positive and significant for all waves, but the impact is weak (Fig 7E).

#### Responses to vaccination and spatially weighted vaccination

The results show that only lags 5 through 7 for the second vaccine dose are significant (Fig 7F). Although the magnitude is relatively small, similar to retail and recreation, the second vaccine dose effectively changed human behaviours. While spatially weighted vaccination has negligible effects (Fig 7G).

#### Control variables

Of the control variables (Fig 7H), only precipitation is positively significant; the more precipitation, the more likely people are to stay at home.

### Comparison between retail and recreation and residential time

Overall, Figs 6 and 7 show that, although the magnitude differs, retail and recreation, and residential human behaviours are quite the opposite impacted by COVID-19-related information during each wave.

### Descriptive statistics and estimated results

The descriptive statistics of the variables used in the estimation and all estimated results for Figs 6 and 7 are presented in A1–A3 Tables in S1 Data.

### Robustness

#### Estimation using Almon lag model

**Fig 8:**
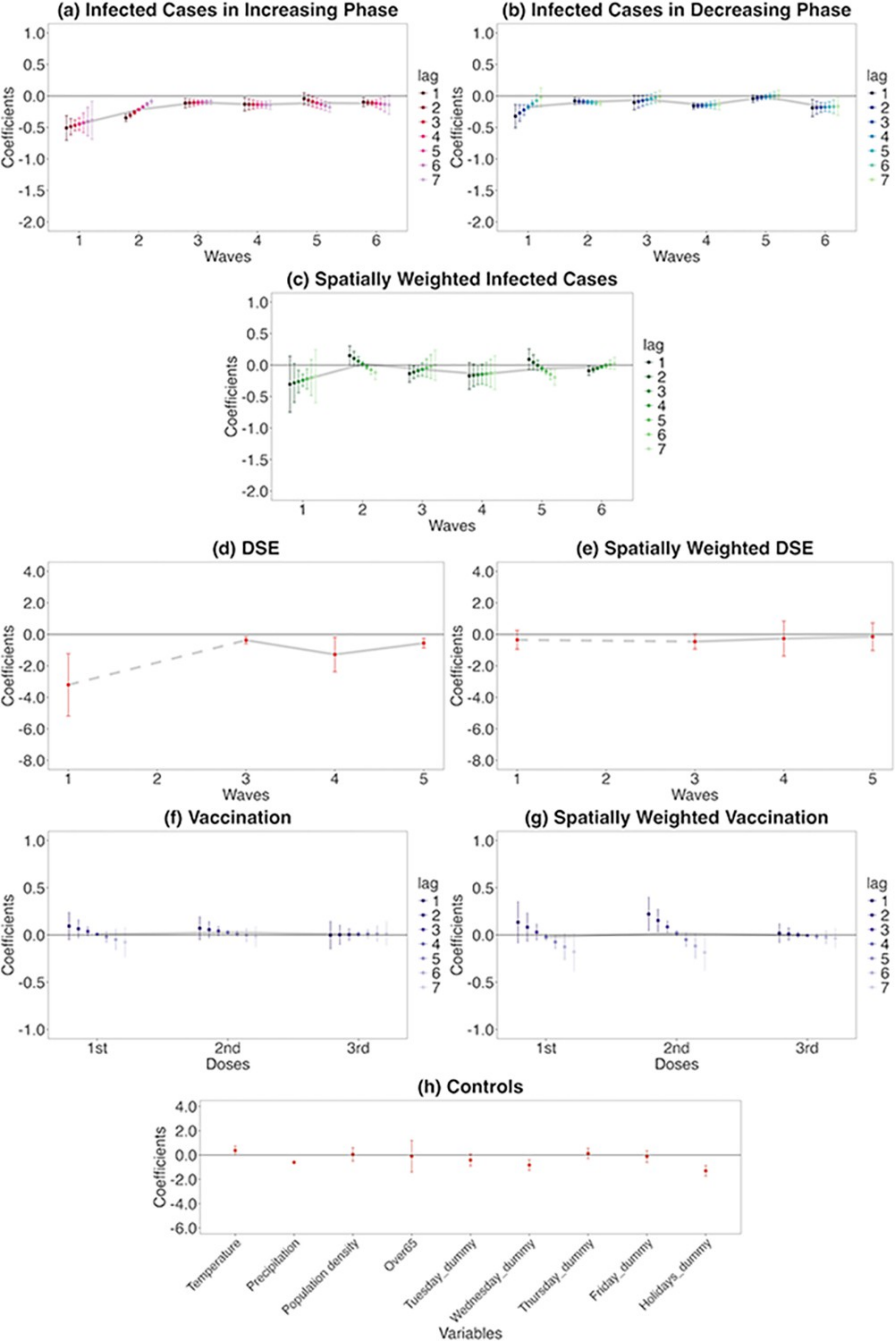
Retail and recreation human mobility responses to COVID-19-related information using the Almon lag model. On each chart, the points are estimated coefficients, and the bars indicate upper and lower 95% confidence intervals. The grey line traces the average coefficients of each lag day. There are six infection waves, but DSEs were only issued for the first, third, fourth, and fifth waves. I take a daily lag from 1 to 7 days for infected cases in the increasing phase, infected cases in the decreasing phase, spatially weighted infected cases, vaccination, and spatially weighted vaccination. I do not take a daily lag for the DSE, spatially weighted DSE, and control variables.

To test robustness, first, I conduct the estimation via the polynomial degree 1 Almon lag model of retail and recreation mobility (Fig 8, detail of the Almon lag model is described in Appendix B in S1 File). As shown in Fig 8, although vaccination is only significant at the second dose lag-4 (and although slightly different results for spatially weighted vaccinations), the results indicate a similar tendency to Fig 6 regarding the response of retail and recreation mobility.

**Fig 9:**
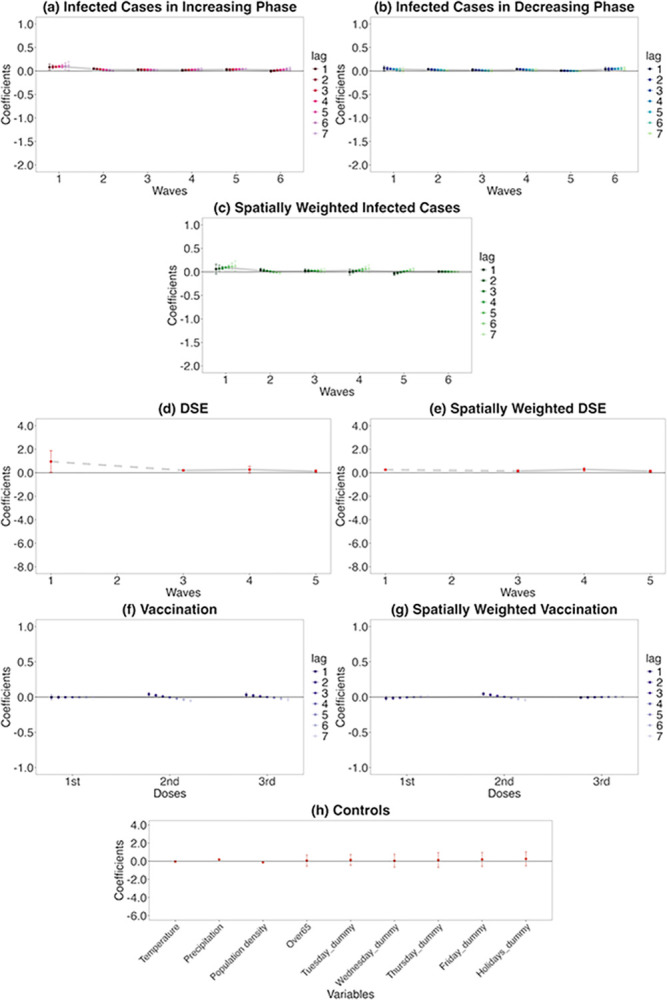
Residential time responses to COVID-19-related information using the Almon lag model. On each chart, the points are estimated coefficients, and the bars indicate upper and lower 95% confidence intervals. The grey line traces the average coefficients of each lag day. There are six infection waves, but DSEs were only issued for the first, third, fourth, and fifth waves. I take a daily lag from 1 to 7 days for infected cases in the increasing phase, infected cases in the decreasing phase, spatially weighted infected cases, vaccination, and spatially weighted vaccination. I do not take a daily lag for the DSE, spatially weighted DSE, and controls.

The results estimated using the Almon lag model for residential time are shown in Fig 9. These results generally support those in Fig 7.

#### Estimation of the extended model

For the second robustness test, I extend estimation model (2) by adding new variables. The reason for extending the model is that other factors may have also affected human mobility during the pandemic. People may be affected not only by the growth rate of infections but also by their absolute number. The number of infected cases tended to increase with repeated waves of infection (Fig 3 and A1 Fig in S1 File). In addition, policy measures other than the DSE were implemented to suppress the spread of infection in Japan. One such measure are the quasi-emergency measures (QEM). The difference between the DSE and QEM is that the DSE is issued at stage four (infection outbreak) of a four-tier alert system, whereas the QEM is issued at stage three (rapid increase in infection), or stage two (gradual increase in infection) in some cases. Furthermore, the DSE is issued at the prefectural level, whereas the QEM is issued at the municipal level [52, 53]. Moreover, the QEM does not require the closure of facilities and stores. The QEM was issued only from the fourth to the sixth waves. The second measure is school closure. Requests for school closure occurred in the first and second waves [54, 55]. The duration of QEM and school closures are presented in Appendix C in S1 File.

In summary, possible missing factors include: 1) the level of the number of infections, 2) QEM, and 3) school closure. To avoid possible omitted variable problems and ensure the robustness of the main estimations above, I also use these variables and their spatially weighted variables to conduct estimations of the extended model. Only for the level of the number of infections, the phases of the infection are separated for the increasing and decreasing. For 1) the level of the number of infections, I use the IHS transformation of newly infected cases of the 7-day backward moving average calculated using [38]. 2) QEM is a dummy variable that takes the value of 1 if a QEM is issued in any municipality within a prefecture, and 0 otherwise. For 3) school closure, a dummy variable is used, which takes the value of 1 if school closure is implemented in a prefecture and 0 otherwise.

All human mobility variables of Google’s COVID-19 Community Mobility Reports, such as *retail & recreation*, *grocery & pharmacy*, *parks*, *transit stations*, *workplaces*, and *residential*, were analysed. D1–D6 Figs in Appendix D in S1 File display all results for the extended model.

*Retail and recreation*. D1 Fig in S1 File shows the results of the human mobility responses in retail and recreation. All coefficients of the variables common to Fig 6 and D1 Fig in S1 Appendix in S1 File are quite similar. Exceptions are the fourth wave of spatially weighted infected cases and the second DSE, which became insignificant.

Focusing on the newly added variables, regarding the level of infected cases in the increasing phase, in all waves, responses are attenuated as the number of lag days increases. Looking at lag day 1 only, the response in wave one is large (-1.16), and the magnitude of the response decreases over waves two (-0.62) and three (-0.27). The response increases again in the fourth wave (-0.39) and subsides slightly in the fifth wave (-0.29), but the response in the sixth wave (-0.35) is at the same level as in the fourth wave. In the decreasing phase, the response decreases with each lag day, similar to the increasing phase. The overall response is smaller during the decreasing phase than during the increasing phase. Again, focusing on lag day 1, the response decreases from wave one to wave three; in wave four, the response is insignificant for all lag days. The coefficient for the fifth wave is small, whereas that for the sixth wave is slightly larger. The spatially weighted levels of infected cases are significant for some lag days of the second wave with negligible coefficients and insignificant for almost all results.

For the QEM, the coefficients for waves four and six are not significant, and although it is significant for wave five, the coefficient is very small (-0.26). However, for spatially weighted QEM, there is a large response in wave four (-1.15). Compared to these, the coefficient for the DSE is -2.39 in wave one and not significant in wave three, and -1.27 in wave four and -0.70 in wave five. On the one hand, the results are insignificant for school closure and spatially weighted school closure.

*Residential time*. The results of the residential time responses are shown in D2 Fig in S1 File. Again, the results for the common variables between the main results (Fig 7) and D2 Fig in S1 File are similar. For the level of infected cases in the increasing phase, the response is large in the first wave and smaller in the subsequent waves. The results for the level of infected cases in the decreasing phase are insignificant; even if significant, the coefficient is minimal. However, a response is observed in the first wave of spatially weighted infected cases. For the QEM, although the results are insignificant for all waves, a slight response is observed in the fifth wave of the spatially weighted variables. None of the coefficients are significant for school closure and spatially weighted variables of it.

*Grocery and pharmacy*. Focusing on the results for grocery and pharmacy human mobility, almost all results show insignificant responses (D3 Fig in S1 File). Slightly significant responses are observed in the first and fourth waves of the level of infected cases in the increasing phase, and the first wave of the level of infected cases in the decreasing phase. In addition, some lag days of the second vaccination dose show a slight increasing response. Finally, some lag days of the second wave of spatially weighted infected cases show significant human mobility changes.

*Parks*. For parks (D4 Fig in S1 File), most of the results are insignificant. The exception is that some lag days of infected cases in the increasing phase are significant, but the magnitude is small. Instead, some lag days of the second vaccination dose show increased mobility responses. In addition, the second wave of spatially weighted infected cases shows significant human mobility changes. For the control variable, precipitation is significantly negative, with a large coefficient.

*Transit stations*. Regarding transit stations (D5 Fig in S1 File), the tendency is similar to that of retail and recreation. For infected cases in the increasing phase, the magnitudes of human mobility responses decay from the first to the third wave. The coefficient tends to be smaller or flat with each lag day during these waves. By contrast, in the fourth to sixth waves, the coefficient becomes significant and larger with each lag day. The responses are smaller during the decreasing phase than during the increasing phase. For spatially weighted infected case, significant results are observed only in the first wave. For the level of infected cases, only the increasing phase shows significant results, with lag 1 day being large. Regarding the DSE, the first and third waves show significantly negative responses. Only the fourth wave of the spatially weighted DSE is significant and negative. However, QEM, school closure, and vaccination show insignificant results.

*Working places*. The last are working places (D6 Fig in S1 File). Almost all coefficients are insignificant. Exceptions are negative responses in the sixth wave of infected cases in the decreasing phase and the first wave in the spatially weighted infected case.

#### Comparison of main estimations and robustness checks

The results of retail and recreation human mobility and residential time of the main estimation (Figs 6 and 7) and robustness tests (Figs 8 and 9 and D1 and D2 Figs in S1 File) indicate a similar tendency. That is, regarding infected cases:

- Decreasing responses from the first to the third waves of the increasing phase
- Augmented responses in the fourth to the sixth waves
- Heterogeneity of responses in increasing and decreasing phases
- Lag day trend
- Spatial spillovers in the first, fourth, fifth, and sixth waves

Regarding DSEs and vaccination:

- Significant responses of DSEs and second-dose vaccinations in own prefectures

## Discussion

**Fig 10:**
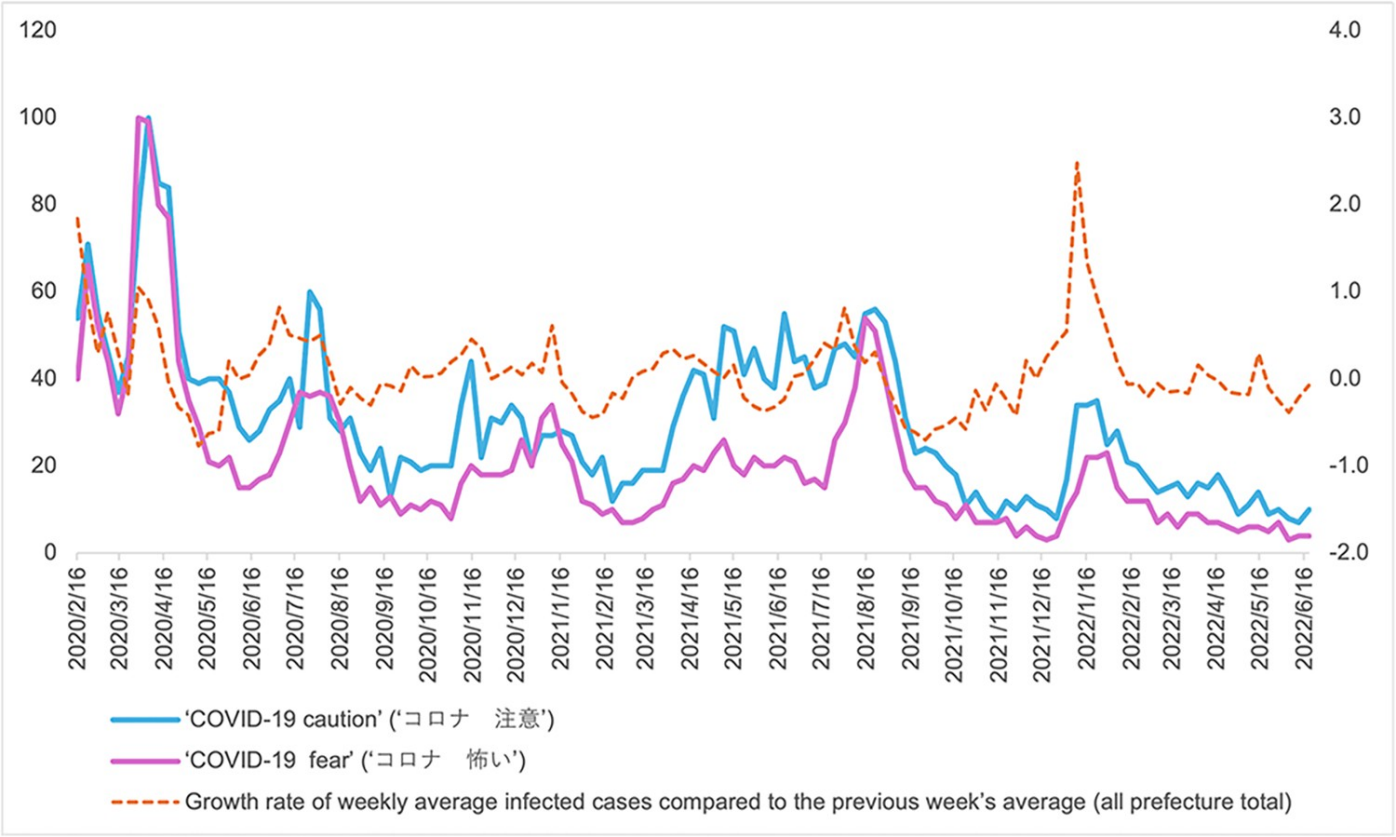
Search results using Google Trends. The search words are ‘COVID-19 fear’ (‘コロナ　怖い’ in Japanese) and ‘COVID-19 caution’ (‘コロナ　注意’ in Japanese) from 16 February 2020 to 19 June 2022 for the whole of Japan using Google Trends data [56] (on the left-hand axis). The growth rate of all prefecture weekly average total infected cases (using data from [57]) compared to the previous week’s average is approximated by the IHS difference (on the right-hand axis).

From the responses of retail and recreation mobility (Fig 6) and residential time (Fig 7), during the increasing (exacerbating) infection phase in the first wave, people largely feared and were cautious about the increase in infection numbers and, thus, largely reduced their outgoing behaviours. These results support the findings of a previous study [12]. To examine the trends in people’s fear and caution in Japan as a whole, I use Google Trends data for the number of searches entered for fear and caution from 16 February 2020 to 19 June 2022 [56]. The search words are ‘COVID-19 fear’ (‘コロナ　怖い’ in Japanese) and ‘COVID-19 caution’ (‘コロナ　注意’ in Japanese). From Fig 10, fear and caution peaked during March–April 2020 in the first wave; in April 2020, the first DSE was issued. During the early stages of the pandemic, fear and caution regarding unknown infections were strong. Incidentally, the reduction in human mobility during the first and second waves weakened each day, as people presumably tended to respond more strongly to the most recent information in the early stages. In addition, the wide CIs of the 6- and 7-day lags in the first wave of the increasing phase indicate that human behaviours on these lag days varied widely among prefectures after receiving infection information.

From the estimation results, people gradually became accustomed to the infections during the first three waves of the increasing phase. This process can be described as ‘habituation’; that is, people become accustomed to similar infection information and gradually decrease their fear and caution. Habituation trends of outgoing behaviours within 2020 have already been recognised in [23, 26]; I also confirm this trend. In addition, as shown in Fig 10, fear and caution rose simultaneously with the increase in infections but gradually diminished compared to the first wave through the second wave in the summer of 2020 and the third wave in the winter of 2020.

By contrast, during the decreasing phase, people may become less fearful and less cautious about the infection. In addition, if human mobility greatly decreases during the increasing phase, a positive rebound may occur during the recovery phase. However, from the results (in Figs 6 and 7), smaller magnitudes of human mobility responses in the decreasing phase compared to the increasing phase over the first three waves exhibited heterogeneous responses. People did not start going out for retail and recreation as before or shortened the residential time spent. This heterogeneity shows that people were cautious about the recovery of human mobility despite habituation trends in the increasing phase, possibly because of fear and caution of COVID-19 (Fig 10). These findings are novel because previous studies did not separate the increasing and decreasing phases of infection; therefore, they did not find such heterogeneity.

**Fig 11:**
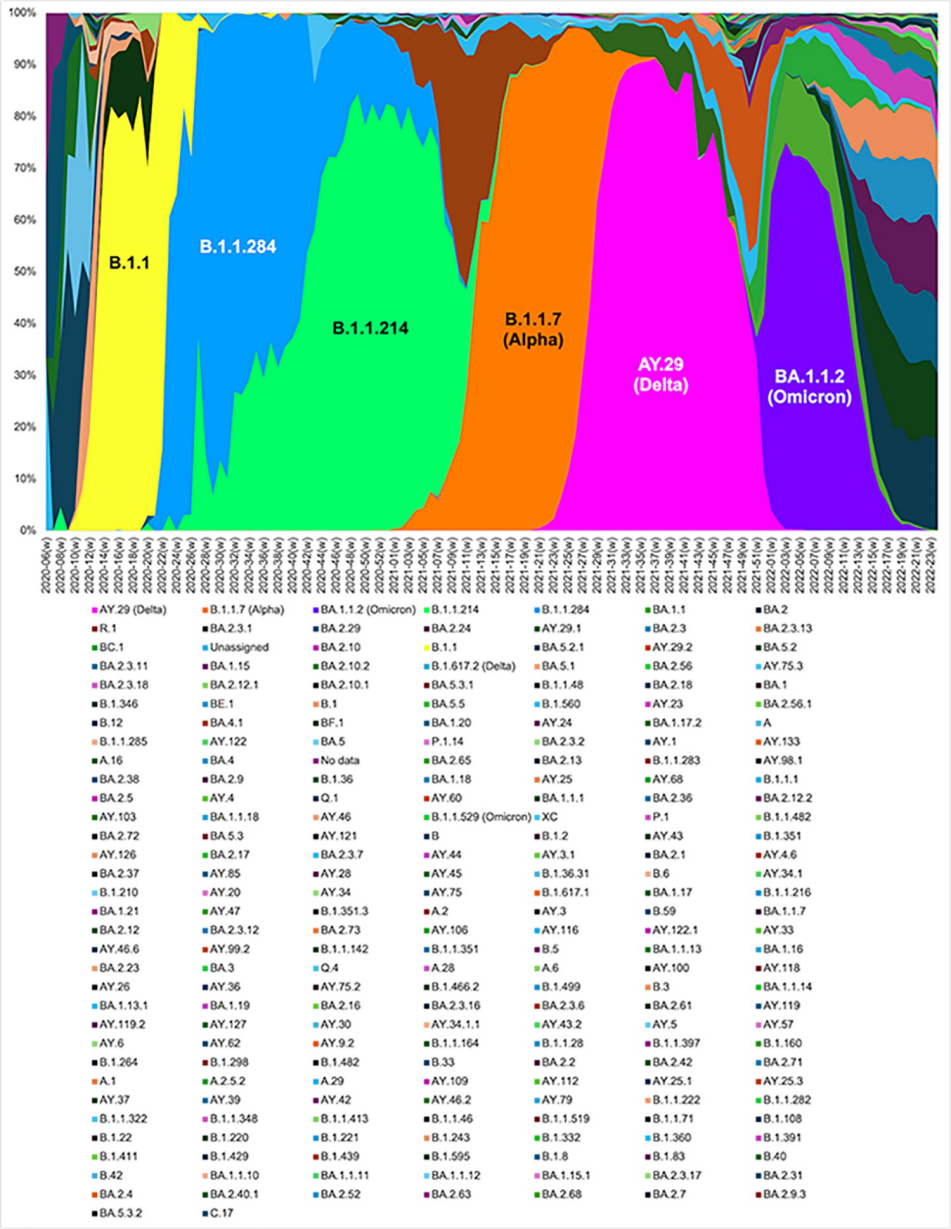
Stacked graph of the weekly trend of the PANGO lineage in the entire Japan. 226 Phylogenetic Assignment of Named Global Outbreak (PANGO) lineages in Japan are displayed using the data obtained from the National Institute of Infectious Diseases [59].

In contrast, from the fourth to the sixth waves (from spring 2021 to June 2022), the Alpha, Delta, and Omicron variants began to dominate, respectively [58], I found a slowdown of habituation. The prevalence of COVID-19 variants is shown in Fig 11. Fig 11 shows the weekly transition of 226 Phylogenetic Assignment of Named Global Outbreak (PANGO) lineages in Japan, obtained from [59]. The first major three, ‘B.1.1’, ‘B.1.1.284’, and ‘B.1.1.214’ prevailed in Japan roughly from April 2020 to March 2021, which corresponds to the first to the third waves in this study. Variants of interest and of concern announced by WHO [60] are the last major three in the figure, ‘B.1.1.7 (Alpha)’, ‘AY.29 (Delta)’, and ‘BA.1.1.2 (Omicron)’. In Japan, the Alpha variant prevailed roughly from March 2021 to July 2021, corresponding to the fourth wave, the Delta variant roughly from July 2021 to the end of 2021, corresponding to the fifth wave, and the Omicron variant roughly from the end of 2021 to April (or May) 2022, corresponding to the sixth wave.

From the estimation results, the responses of people during the increasing phase, which had declined until the third wave, began to strengthen again and the habituation level decreased. Each new variant exacerbated the speed of infectivity [61], which led to increased fear and caution. In addition, unlike in the first three waves, where the response weakened daily or remained flat, from the fourth to the sixth wave, the responses tended to intensify from lag day 3 to lag day 5 in each wave. Due to the emergence of new variants, people probably became much more reluctant to go out after receiving information about growing numbers of infections for successive days beyond their anticipation. These two findings–the slowdown of habituation, and intensification of the response from lag day 3 to lag day 5 from the day when people receive information–are new to the literature.

**Fig 12:**
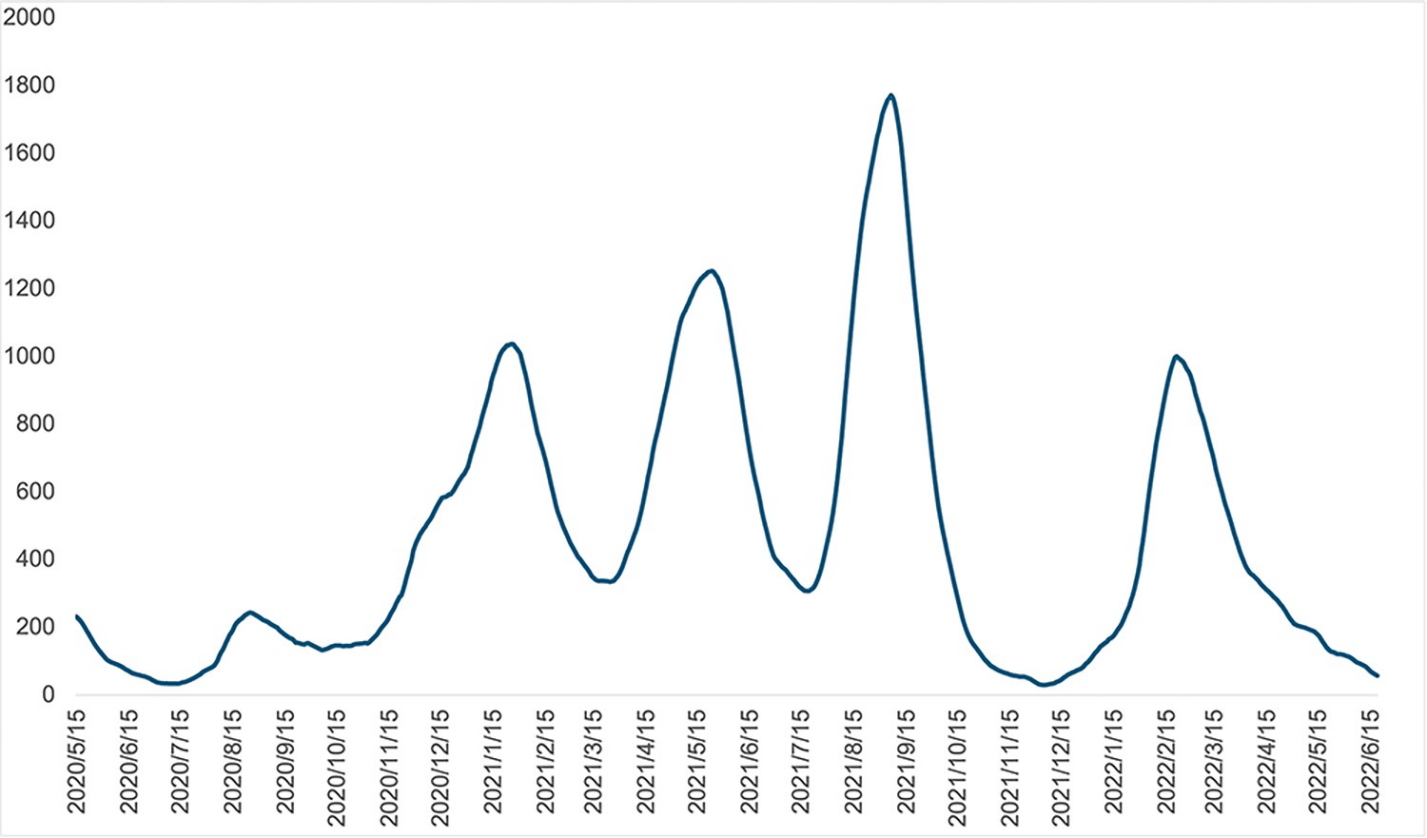
Number of severe cases using 7-day backward moving average of all prefecture totals. Due to data limitations, the graph starts from 14 May 2020. The figure is constructed using the data obtained from the Ministry of Health, Labour and Welfare [57].

Fig 12 shows the number of severe cases using a 7-day backward moving average for all prefectures, obtained from [57], to confirm the above results. As shown in the figure, the number of severe cases increased in the spring of 2021, during the fourth wave, when the Alpha variant prevailed. A further increase was witnessed from the summer to autumn of 2021, in the fifth wave, when the Delta variant prevailed. However, during wave six, the time when the Omicron variant prevailed, the number of severe cases had stabilised at the same level as in wave three. In addition, as shown in Fig 10, people’s fear and caution increased again from April 2021 to August 2021, when the Alpha variant prevailed and when the Delta variant started to prevail. This may reflect people’s reactions to new, unknown variants.

Interestingly, the decreasing phase of infection in wave four suggest a prominent recovery of human mobility, which again implies the heterogeneous response between the increasing and decreasing phases. This recovery is probably due to some positive events: the promotion of a rapid vaccination programme [17, 18] and new information regarding COVID-19 treatment and the post-infection situation [19, 20]. Referring [19], which was published on 7 December 2021, a brief list of treatments is as follows: recommendation for anti-viral drugs and neutralising antibody preparation would be a) Remdesivir (published 20 November 2020), b) Lopinavir/ritonavir (published 17 December 2020), c) Hydroxychloroquine (published 17 December 2020), d) Ivermectin (published on 31 March 2021), e) Tocilizumab or sarilumab (IL-6 receptor blockers) (published on 6 July 2021), and f) Casirivimab and imdevimab (monoclonal antibodies) (published on 24 September 2021). Referring [20], which was published on 16 October 2021, the outline for a post-infection situation is: ‘Although most people with COVID-19 experience mild symptoms or moderate illness, approximately 10–15% of cases progress to severe disease, and about 5% become critically ill. Typically, people recover from COVID-19 after 2 to 6 weeks. While most people with COVID-19 recover and return to normal health, some people can have symptoms that last for weeks or even months after recovery from acute illness though they are not infectious to others during this time’; ‘Anyone with COVID-19 can get post COVID-19 condition, irrespective of the severity of disease’; ‘Common symptoms include fatigue, shortness of breath and a decline in mental abilities like memory or brain fog (cognitive dysfunction)’. Such announcements reduced people’s concerns about COVID-19, which were initially unknown infections, to some extent.

By contrast, in the fifth wave, the estimates are not significant in the decreasing phase, likely because the Delta variant is more transmissible and severe than the Alpha variant in the fourth wave (Fig 12) [61], making people more fearful and cautious. Again, from Fig 10, around August 2021, ‘caution’ rises further and ‘fear’ shows a spike. Therefore, human mobility did not recover. In the sixth wave, the Omicron variant is prevalent and more transmissible than the previous ones (see growth rate of infected cases around January 2022 in Fig 10), but less severe (Fig 12) [61]. Therefore, people probably went out more when the number of infections decreased. From Figs 10 and 11, the Omicron variant had started to prevail between the end of 2021 and the beginning of 2022, and ‘caution’ and ‘fear’ rose, but not as much as in the Alpha and Delta prevailing periods. After going through high-severity variant strains, people quickly recovered their going-out behaviour when less severe variants started to subside. In a sense, this could also be a sign of habituation.

With respect to vaccination, positive estimates are expected for retail and recreation, and negative ones are expected for residential time because, as the vaccination rate increased from the previous week, the more secure people felt about going out. The effect of vaccination is apparent: increased second-dose vaccination rates led to the recovery of human mobility. At that time, the Japanese government released information on the efficacy of the second dose [62]. During the long-term COVID-19 pandemic, a rapid vaccination programme and its information dissemination help alleviate people’s concerns regarding COVID-19 infection and make them feel safe and secure. However, because the timing of the third vaccination varied greatly by person, human mobility does not show substantial recovery. These findings support the positive effects of vaccination on mobility, as verified in the Appendix in S1 File of [28]; however, the novelty of this study is that vaccination doses are separated, as are the different responses per dose.

As for the responses to DSEs, from the results, it is more plausible to consider changes in the human mobility response as per varying requests rather than habituation (see Details of DSEs in Appendix A in S1 File). Since the first DSE (first wave) was a very strong request, it greatly reduced the retail and recreation human mobility. Additionally, the mobility reduction was large in the third DSE (fourth wave), in which the requests were strengthened compared to the second DSE to some extent. In comparison, the magnitudes of the responses in the second and fourth DSEs (third and fifth waves) of retail and recreation, in which the requests were somewhat mitigated, are low. This result differs substantially from the findings of similar studies [27, 28]. Specifically, [27] finds that as the number of DSE increases, the magnitude of the negative relationship between going-out behaviour and DSE decreases. In addition, [28] concludes that a repetition of the DSEs becomes ineffective and less compliant with social distancing behaviour over time. Further, [63] shows that repeated DSE decreased mental and physical symptoms overall. Conversely, the results of this study do not imply a habituation for repeated DSEs, but rather a response for the relief from the first to the second, the stricter from the second to the third and the relief from the third to the fourth DSEs, as per Details of DSEs in Appendix A in S1 File. This difference is probably due to differences in the estimating equations. First, in the model, I consider spatial spillovers that represent human travel between prefectures on human mobility using a spatial weight matrix. Second, the estimation model considers interactive effects. On the one hand, the responses of residential time regarding DSE are trivial.

This study is the first to demonstrate that responses to information about COVID-19 infection also arise from cross-prefectural travel. These spatial spillovers were remarkable when the public feared and were cautious about the new infectious disease and the emergence of its more infectious variant strains (in the first and fourth waves). The wider CI during the first to the fourth waves of spatially weighted infected case of retail and recreation mobility (Fig 6c) than the response for one’s own prefecture (Fig 6A and 6B) indicates that the impacts from other prefectures vary greatly by prefecture.

However, there is no evidence of DSE and vaccination spatial spillovers; this is likely because both are only valid in one’s own prefecture. Unlike infections, the DSEs issued in some prefectures do not spatially propagate to others. Suppose a DSE is issued in a neighbouring prefecture with high mutual movement, but the DSE remains inactive in its own prefecture; as such, people in the own prefecture do not stop going out. A few people may be cautious about the DSE in neighbouring prefectures, but this is not sufficient to significantly reduce human mobility. However, once the DSE is issued in its own prefecture, people start to refrain from going out. This lack of spatial effects in PHIs is also observed in another study [8], which finds that the spatial spillover of PHIs to neighbourhoods has only limited effects on human mobility. Similarly, vaccinations in other prefectures do not spatially propagate to their own prefectures. In the present analysis, the effect of an increase in human mobility by vaccination is believed to be due to individual vaccination; the significant responses to the vaccination rates in their own prefectures probably reflect this. Therefore, an increase in the number of vaccinated people in neighbouring prefectures does not motivate people in their own prefectures to go out.

The results of the first robustness check–the Almon lag model estimation–confirmed the above main findings. Regarding the results using the extended model, for the increasing phase of the level of infected cases in retail and recreation mobility, a habituation trend was observed from the first to the third waves, as in the main estimations. The decreasing phase showed smaller responses, which again indicates people’s fear and caution. A significant response in residential time in the first wave of the level of infected cases also represents people’s fear and caution. These responses are common to the result for the infected cases which approximate the growth rate of the level of infected cases. By contrast, responses for the QEM are insignificant, except that the spatially weighted QEM has a large response in wave four for retail and recreation. This is because QEMs are less demanding of curfews than DSEs. Significant results in the spatially weighted QEM of the fourth wave probably represent people’s reactions to new behavioural restraint requests that were first issued in other prefectures. The insignificant results of school closures for both retail and recreation and residential time are probably due to the fact that a request was not for all citizens.

There are other findings for other mobility categories. The insignificant response of almost all estimated results of grocery and pharmacy mobility reflects the fact that, even during the DSE period, local governments did not ask to restrict outings to maintain the necessities of life, such as groceries and pharmacies (e.g. [64, 65]). Requests from local governments were based on the Act on Special Measures for Novel Influenza. Article 11 of the enforcement order of the Act [66], which sets out the stores, etc., subject to requests for restrictions on use, etc. (due to the prevalence of infections), states that ‘restrictions exclude sales floor that provides food, medicines, medical equipment and personal protective equipment, other sanitary goods, regenerated medical products, or fuel, and in addition, other goods specified by the Minister of Health, Labour and Welfare as indispensable for daily life’. Therefore, people did not substantially change their outings to these stores, with or without DSEs. In other words, human mobility in the grocery and pharmacy did not change largely even when infection was exacerbated or when DSEs were issued.

Most of the results for parks are insignificant. No restriction requests were issued for parks, subject to the condition that people avoid gathering in close proximity, as stated in the Prime Minister’s press conference under the first DSE at the time [67]. During the DSE, especially the first DSE, many people went to play in the park from a social distance perspective [68]. The CIs of the estimated results for parks are large, probably due to the fact that people were divided on whether to refrain from going out, including parks or to go to parks.

Transit stations mobility shows a similar tendency as retail and recreation mobility, such that habituation occurred from the first to the third waves in the increasing phase of infected cases, smaller responses in the decreasing phase than in the increasing phase, and significant results in the first wave of the spatially weighted infected cases. This is because transit stations often have large commercial and downtown areas in their hinterlands, and many people heading to retail and recreation use transit stations. Of course, many people who go to work also use transit stations, but the fact that fluctuations in the mobility of working places can be ignored (as stated below) suggests that the majority of the changes in transit stations might have originated from retail and recreation mobility. For the results for working places, no major movements were observed. This is likely because even during DSE, although the use of teleworking was encouraged, commuting to work was not restricted [64, 65].

In summary, retail and recreation mobility were the most responsive to infections, DSEs, and vaccinations. The government asked people to refrain from unessential outings, and the results for retail and recreation reflected this. It is also likely that many people believed that these unessential places were more likely to spread the infection and, thus, refrained from going there when the infection increased.

## Conclusions

I acknowledge that this study possesses the following limitations:

1. The data, which are macro data aggregated by prefecture, do not uncover the diversity of behaviours within a prefecture or for each individual.
2. Although data on each variant’s spread status, infection speed, and severity are crucial for the human mobility response, I cannot incorporate this information into the estimation model because of a lack of these prefectural data. Human mobility responses to these pieces of information are most likely included in the responses for the infected cases.

Even considering the limitations noted above, the results of this study have several key policy implications. If it becomes necessary to control human mobility during a future pandemic, flexible policies monitoring human mobility responses to information are desirable. One prominent response is that, under repeated waves of similar infection, a larger-than-expected going-out behaviour is expected due to people’s habituation. In addition, the infection rate in other regions, which also affects human mobility, should be considered. If a region has high movement with other regions that exacerbated the infection situation, the residents of that region have reduced their outings, and vice versa. In addition, people show different reactions to the increasing and decreasing phases; therefore, if the infection starts to reduce, it is important for policymakers to monitor whether there is a rapid or mild recovery in human mobility. Additionally, a rapid vaccination program could alleviate people’s concerns.

In Japan, people appear sensitive to the intensity of human mobility control measures. Thus, the severity of the requests for human mobility control is also expected to have an announcement effect from the government. Therefore, fine-tuning policies of mobility control is possible. Implementing flexible human mobility control policies by precisely monitoring human mobility can thus prevent excessive or insufficient mobility control requests. Such a flexible policy could efficiently suppress infection spread and prevent economic activity reduction more than necessary. On the one hand, the estimated results show that DSEs issued in other prefectures, even with high levels of mutual movement with their own, do not suppress mobility in their own prefectures. Therefore, exacerbated infections in prefectures where DSEs have not been issued could propagate to prefectures where DSEs have been issued, thus reducing the efficiency of DSEs. Hence, it is more efficient to coordinate the issuance of DSEs as much as possible between prefectures with high levels of mutual movement to efficiently suppress human mobility. Alignment among local governments is important.

These implications are useful for EBPM during future pandemics. The Japanese government announced its ‘Government Action Plan for Pandemic Influenza’ in April 2024 [69]. According to the Plan, ‘the impact on people’s livelihoods and socio-economic activities will be reduced by switching measures to prevent spread flexibly and efficiently in response to changes in the situation’, which is the same as this study’s implication encouraging flexible policy. It also mentions that ‘measures are taken for areas, periods, and business types within the minimum necessary’. The plan also considers the long duration of the pandemic: ‘given the possibility of a prolonged pandemic, the measures to be taken to deal with multiple waves of infection and the flexible switching of measures in response to the promotion of vaccination programs and therapeutic agents should be taken’. This study found that human responses changed through the long-term pandemic and that these pieces of information (vaccination programs and therapeutic agents, etc.) changed human mobility responses. And the plan also states that ‘policies will be implemented based on the EBPM approach, which utilises information, statistics and other data that have important relevance for measuring policy effectiveness’. This study supports the government’s future pandemic policies and could be one of the cornerstones of policy promotion based on EBPM.

## Supporting information

S1 File (PDF)

S1 Data Appendix tables. (XLSX)
