# Supplementary material for: Unravelling habituation for COVID-19-related information: A panel data study in Japan
Source: PLoS One. 2024 Jul 30;19(7):e0306456. doi: 10.1371/journal.pone.0306456 (PMC11288468; doi:10.1371/journal.pone.0306456)
Supplement: S1 File — (PDF) [file pone.0306456.s001.pdf]

Supplementary Information

Unravelling habituation for COVID-19-related information: A panel data study in Japan

Shinya Fukui*

* Graduate School of Economics, Osaka Metropolitan University, Naka, Sakai, Osaka, JAPAN. (E-mail: shinya.fukui.econ@gmail.com).

Appendix A Further data descriptions

Details of DSEs

PHIs in Japan

Regarding PHIs in Japan, lockdowns accompanied by legal enforcement have not been implemented. Instead, Japan implemented DSEs that requested that people refrain from leaving their residences except for essential and urgent purposes and that facilities shorten their hours of operation or temporarily close down. As such, the Japanese policies for controlling the spread of COVID-19 are referred to as 'soft lockdowns' [1] or 'voluntary lockdowns' [2, 3]. This means that even if a DSE is declared, individuals are not legally regulated but are requested to stay at home; accordingly, as Yabe et al. [4] reveal, going out behaviours are voluntary. To account for this, I consider DSEs by the Japanese government to be COVID-19-related information.

Requests regarding DSEs

The content of requests regarding DSEs is entrusted to each prefecture and varies by prefecture. This section provides an overview of the details of such requests, focusing on Tokyo and Osaka, Japan's two representative major cities. Since both are large cities, the requests were almost identical.

In the first DSE (first wave), the closure of facilities and stores was requested in principle for amusement facilities, universities, tutoring schools, educational (school) facilities, exercise facilities, theatres, gathering and exhibition facilities (including museums), and commercial facilities (including department stores). In principle, the government asked that events be held without spectators.

In the second DSE (third wave), the government asked facilities and stores to reduce their operating hours instead of closing entirely. The maximum attendance for events was set at 5,000 people, easing the limitation imposed on the first DSE.

The third DSE (fourth wave), in which the Alpha variant was prevalent, resulted in a new request to close restaurants and stores serving alcoholic beverages and offering karaoke. In addition, requests to close facilities and stores with footprints of over 1,000 square metres were issued to amusement facilities, exercise facilities, theatres, commercial facilities (including department stores), and museums. Additionally, the government asked that events that attract visitors be held without spectators and that college classes be conducted online. Thus, with the emergence of the Alpha variant, the requests were strengthened.

In the fourth DSE (fifth wave), the government asked restaurants and stores serving alcoholic beverages and offering karaoke to close again. Meanwhile, facilities and stores were not requested to close in principle but were asked to shorten their operating hours. Moreover, the maximum attendance for events was again set to 5,000 people. Thus, these requests were less restrictive than those made during the third declaration.

During all the DSEs, restaurants were asked to shorten their operating hours, and people were asked to avoid unnecessarily leaving their homes. The above information regarding the DSEs was taken from the Tokyo Metropolitan Government [5] and the Osaka Prefectural Government [6].

The first DSE was issued to all 47 prefectures. In contrast, the subsequent DSEs were only issued to some prefectures based on the local severity of infections; specifically, the second DSE was issued in 11 prefectures, the third in 10 prefectures, and the fourth in 21 prefectures. Moreover, the length of the DSE periods differed by prefecture. One exception is that, only in Okinawa Prefecture, DSEs corresponding to the third and fourth were issued without interruption. In all cases, the government asked facilities and stores to close only in the first and third DSEs. In short, the first and third requests were typically strict, and the second and fourth requests were typically lenient; across prefectures [7, 8].

Reasons for utilising week-on-week vaccination rates

There are also reasons, in addition to the ones mentioned in the main manuscript, for utilising week-on-week vaccination rates rather than daily vaccination rates data in the estimations. Since vaccinated people have more reassurance that they can avoid infection, I reason that higher vaccination rates are likely to correlate with a higher portion of people going out in each prefecture. In the extreme, each vaccinated individual will shift from the fearful group (unvaccinated group) to the reassured group (vaccinated group). The size of the reassured group will thus increase in step with the promotion of vaccination in the prefecture, and the going-out behaviour of the group will also increase proportionally. Thus, one might think that the better choice would be to use the daily vaccination rate itself, which ranges from 0–100%.

However, I am using week-on-week differences in human mobilities and residential time as the dependent variables in the estimation. Since fear is expected to decrease considerably immediately after vaccination, the growth from the previous week in going-out behaviour is expected to increase only at this point. In other words, individuals who

already feel reassured and engage in going-out behaviour right after being vaccinated will essentially not contribute to later growth in going-out behaviour. Therefore, I use the difference in the vaccination rate compared to the previous week (incidentally, when I use the daily vaccination rate itself instead, none of the results would have been significant). Theoretically, each vaccinated person shifted from the fearful group to the reassured group and contributed to the growth in going-out behaviour at that point. For example, in the fake data in Fig 1 in the main manuscript, we can see that the number of people going out from 1 June to 8 June 2021 increased by 0.1 million. If we hypothesise that this increase is entirely due to vaccination (and if we ignore the 1 to 7 daily lags used in the estimation), then we can assume that the increase only happens from 1 June to 8 June 2021 and that the 0.1 million newly vaccinated people who joined the reassured group at this time will continue to go out and not contribute to later increases in going-out behaviour.

The other reason I use the difference from the previous week was the multicollinearity between the first and second vaccination doses. Japan's vaccination rate accelerated in 2021 [9, 10]. At the time, due to the Ministry of Health, Labour, and Welfare announcement that two doses of the COVID-19 vaccine can prevent infection [11], most people who received a first dose also received a second dose rapidly. Therefore, the first and second vaccination rates almost perfectly correlate (correlation coefficient = 0.99).

Time series plots of main variables

The time series plots of variables for all prefectures used in the estimation are shown in Fig A1. For human mobility, I use retail and recreation as a typical example. From the figure, infections and human mobility are moving in opposite directions. Further, the first and second vaccinations proceeded rapidly. Additionally, the timing of the DSE differs by prefecture. Prefectural governments decided to request DSEs based on the infection situation in their respective prefectures. DSEs have been issued three or more times in urban areas with high infection cases, such as Tokyo, Kanagawa, Saitama, Chiba, Aichi, Osaka, Hyogo, Kyoto, Fukuoka, Hiroshima, and Hokkaido prefectures, and surrounding urban areas such as Tochigi, Gifu, and Okayama prefectures. Okinawa also showed higher infection cases. By contrast, suburb prefectures, other than those listed above, showed lower infection cases. As previously mentioned, the first DSE was issued for all prefectures.

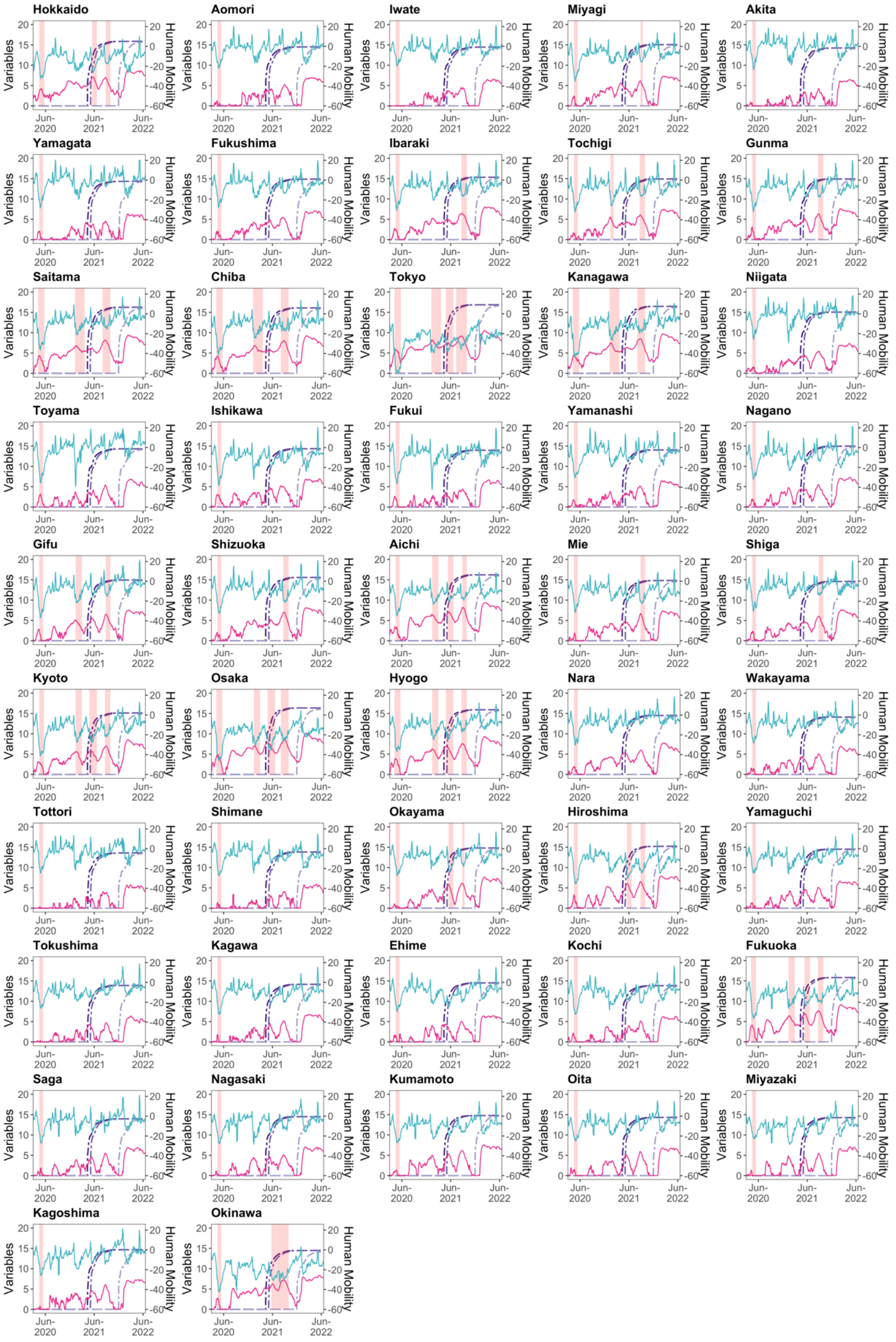
Hokkaido
Aomori
Iwate
Miyagi
Akita
Yamagata
Fukushima
Ibaraki
Tochigi
Gunma
Saitama
Chiba
Tokyo
Kanagawa
Niigata
Toyama
Ishikawa
Fukui
Yamanashi
Nagano
Gifu
Shizuoka
Aichi
Mie
Shiga
Kyoto
Osaka
Hyogo
Nara
Wakayama
Tottori
Shimane
Okayama
Hiroshima
Yamaguchi
Tokushima
Kagawa
Ehime
Kochi
Fukuoka
Saga
Nagasaki
Kumamoto
Oita
Miyazaki
Kagoshima
Okinawa
Variables
Human Mobility
Jun-2020
Jun-2021
Jun-2022

Fig A1. Time series plots of the variables per prefecture used in the estimation. On each chart, the blue-green-coloured line is the 7-day backward moving average using the geometric mean of human mobility in retail and recreation (on the right-hand axis); the red-purple-coloured line is the IHS transformation of the 7-day backward moving average number of infected persons (on the left-hand axis); the purple-coloured double-dashed lines are the IHS transformation of the cumulative number of people vaccinated with 1–3 doses (on the left-hand axis), and the pink-shaded areas are the DSE periods. The data transformation employed here, such as the 7-day backward moving average, is only for visualisation purposes; I use other transformations in the estimation. The figure is constructed using data from [12-15].

Appendix B Performing the estimation through the Almon lag model

When considering the estimating model with lags from 1 to 7,

$$y_{it} = a + \sum_{p=1}^{7} b_p x_{it-p} + e_{it}, \text{(A1)}$$

where y is a dependent variable, x is an explanatory variable, i is a cross-sectional index, t is a time index, p is a lag taken for the time dimension t, a and b are parameters, and e is an error term, the expression of the polynomial degree 1 Almon lag model will be

$$y_{it} = a + \theta_0 \sum_{p=1}^{7} x_{it-p} + \theta_1 \sum_{p=1}^{7} p x_{it-p}. \text{(A2)}$$

θ_0 and θ_1 are to be estimated. The estimated coefficients of parameter b of each lag p can be retrieved from

$$\hat{b}_p = \hat{\theta}_0 + \hat{\theta}_1 p. \text{(A3)}$$

To find the standard errors of the estimated coefficients, I use the following equation:

$$s.e.(\hat{b}_p) = \sqrt{Var[\hat{b}_p]} = \sqrt{Var[\hat{\theta}_0 + \hat{\theta}_1 p]}$$

$$= \sqrt{\sum_q p^{2q} Var[\hat{\theta}_q] + 2 \sum_{q<r} p^{(q+r)} Cov[\hat{\theta}_q, \hat{\theta}_r]}, \text{(A4)}$$

where q and r are the order of θ, that is, the degree of the Almon lag, and $q \neq r$. For a detailed explanation of the Almon lag model, see, for instance, Chapter 17 in [16].

Appendix C DSE and school closure periods by prefecture

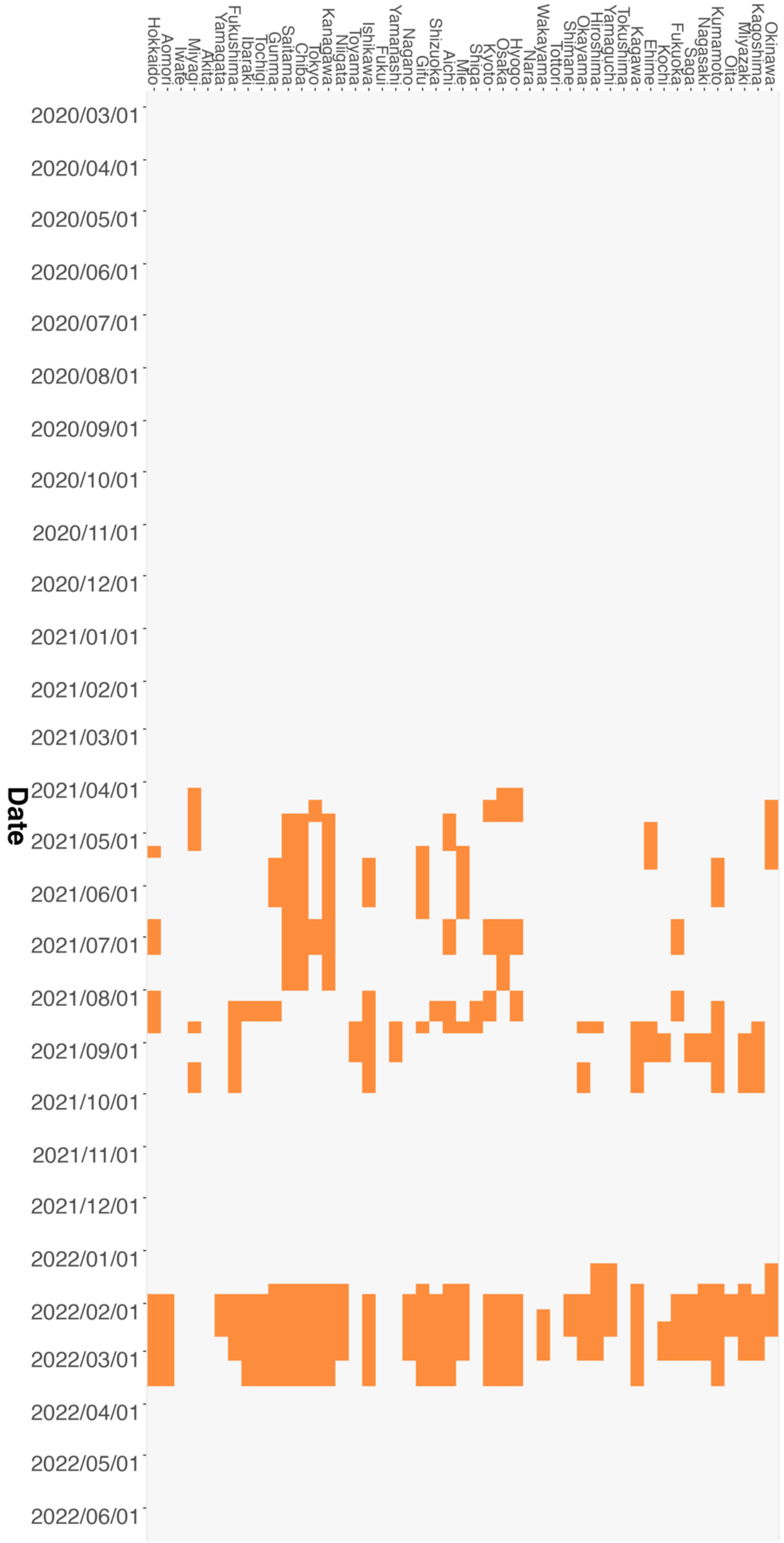

Fig C1. QSE periods for each prefecture. The orange colour indicates the periods over which the QSE was issued.

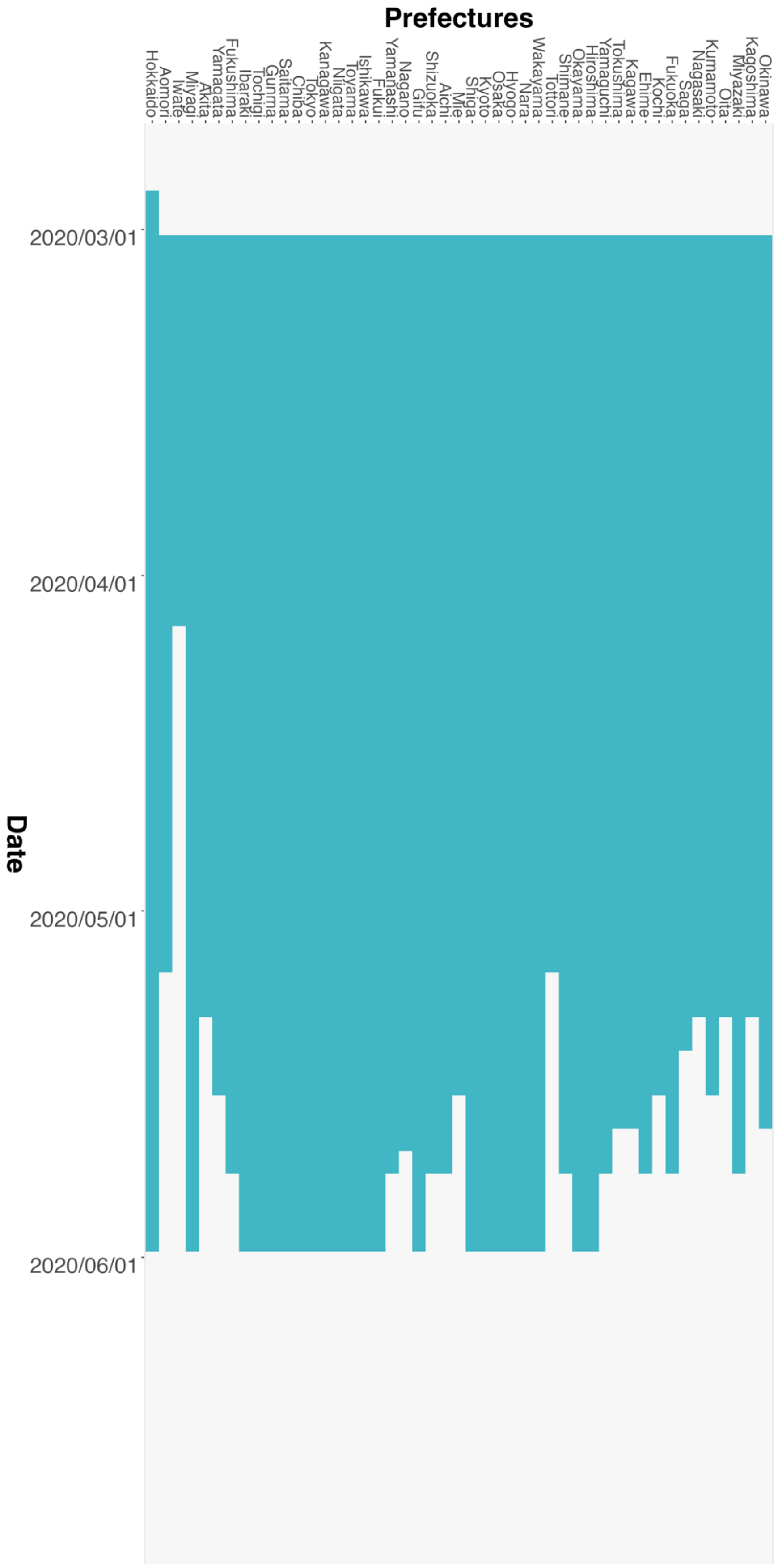

Fig C2. School closure periods for each prefecture. The blue-green colour indicates the periods over which the school closure was issued.

Appendix D Results using the extended model

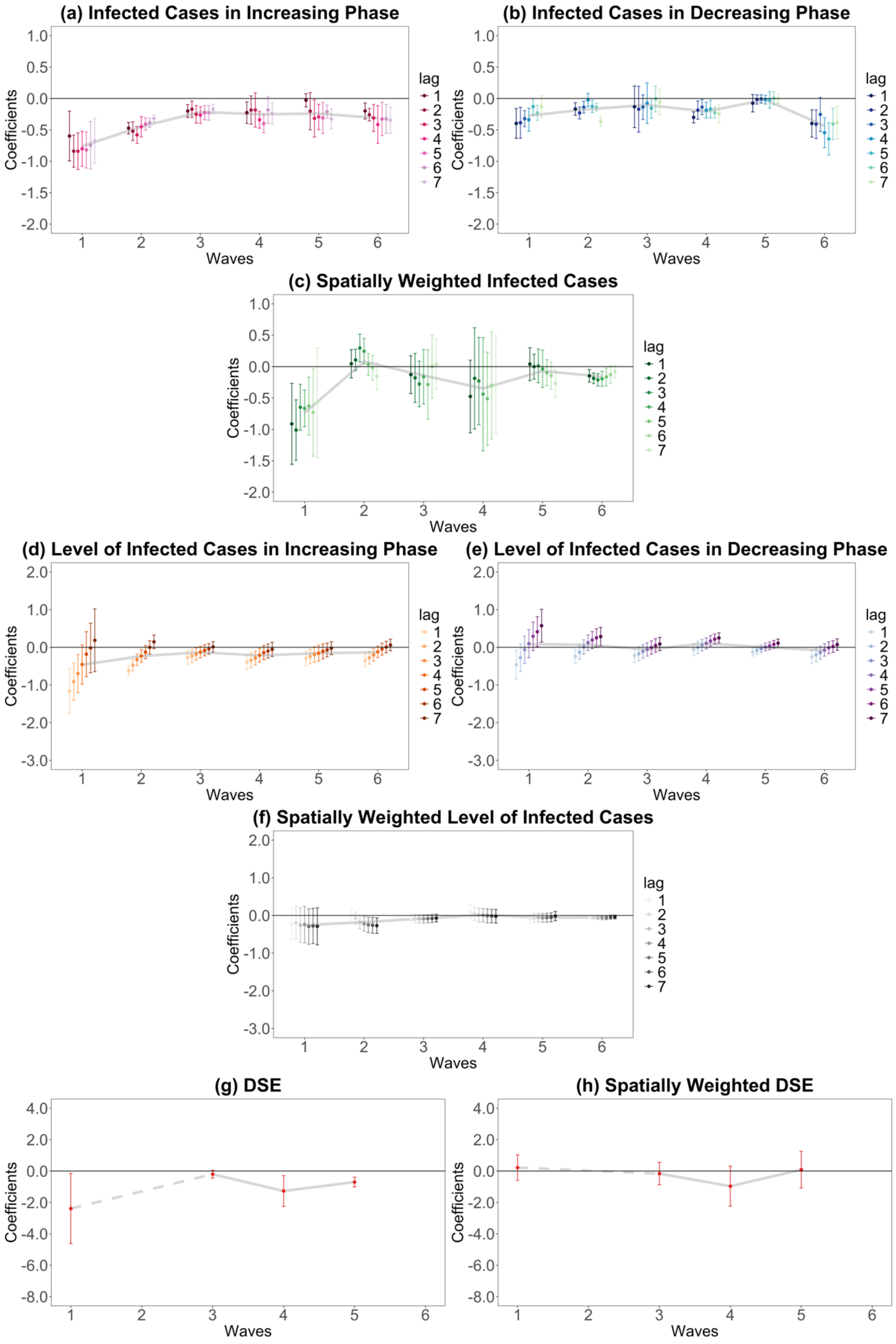

(Cont'd)

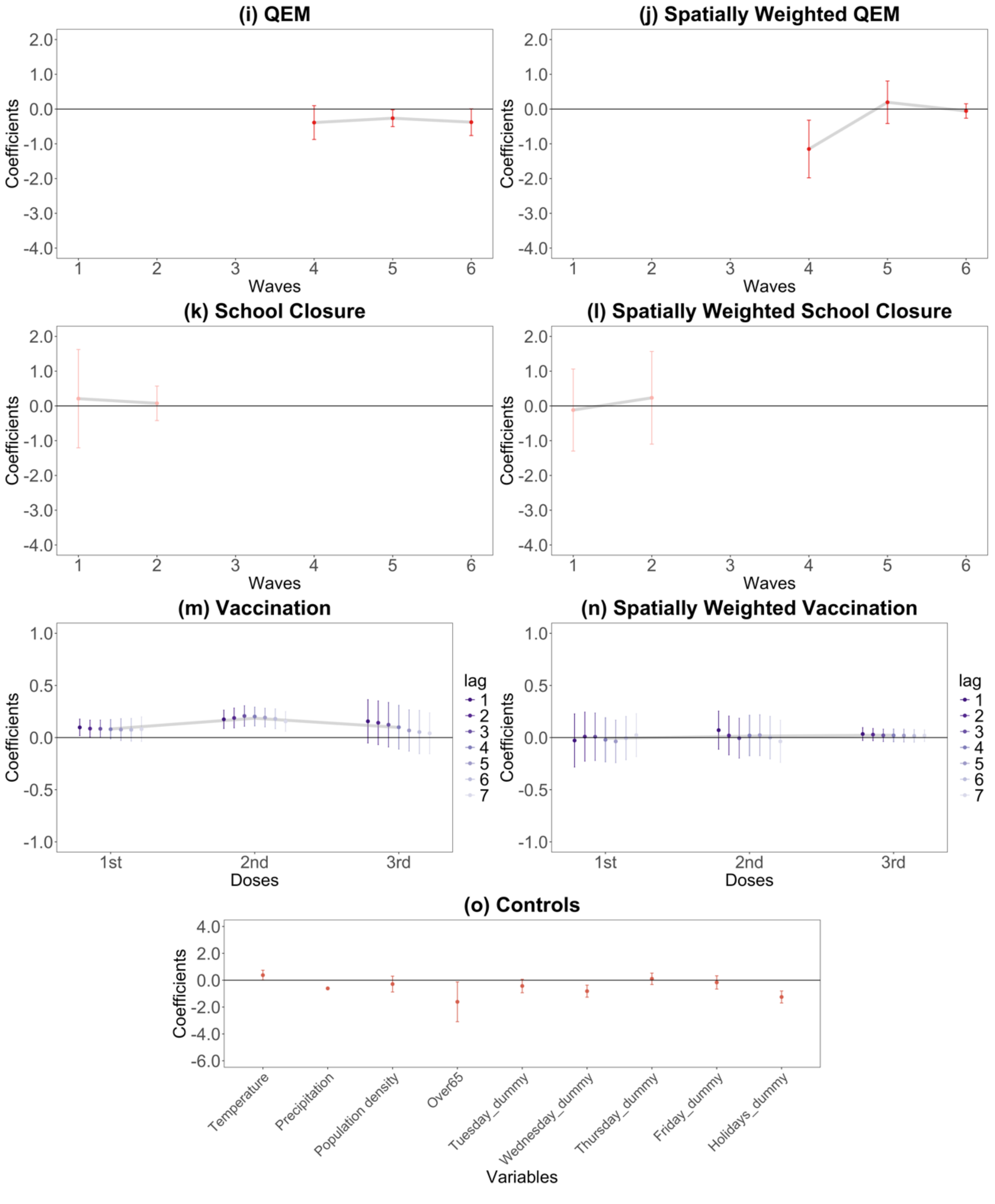

Fig D1. Retail and recreation human mobility responses to COVID-19-related information (extended model). On each chart, the points are estimated coefficients, and the bars indicate upper and lower 95% confidence intervals. The grey line traces the average coefficients of each lag day. There are six infection waves, but DSEs were only issued for the first, third, fourth, and fifth waves; QEMs were only issued for the fourth, fifth, and sixth waves; and school closures were only requested for the first and second waves. I take a daily lag from 1 to 7 days for infected cases in the increasing phase, infected cases in the decreasing phase, spatially weighted infected cases, level of infected cases in the increasing phase, level of infected cases in the decreasing phase, spatially weighted level of infected cases, vaccination, and spatially weighted vaccination. I conduct the regression analysis seven times, from lags 1 to 7. I do not take a daily lag for the DSE, spatially weighted DSE, QEM, spatially weighted QEM, school closure, spatially weighted school closure, and control variables; these estimates are from the lag-1 regression.

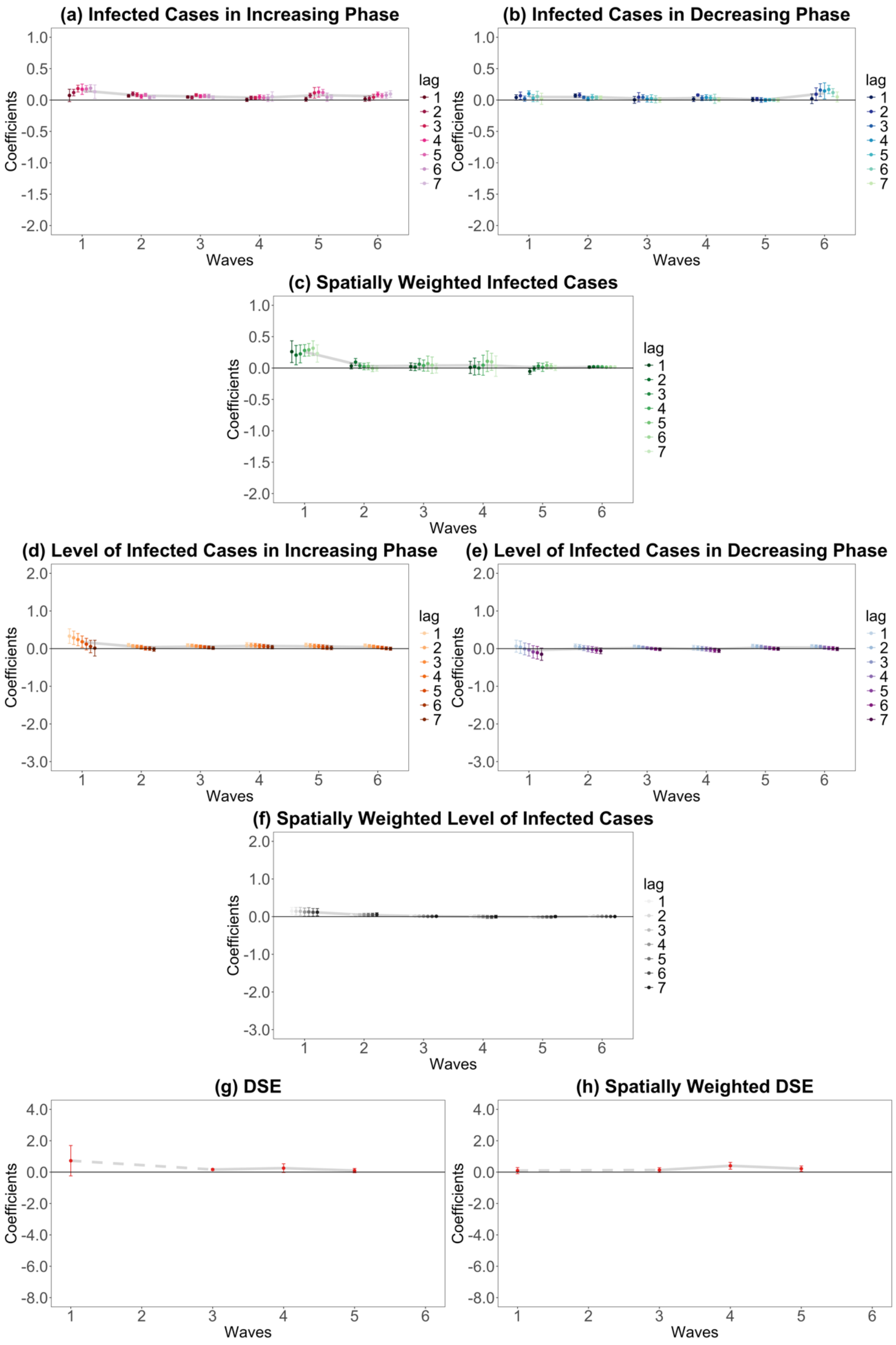
(a) Infected Cases in Increasing Phase
(b) Infected Cases in Decreasing Phase
(c) Spatially Weighted Infected Cases
(d) Level of Infected Cases in Increasing Phase
(e) Level of Infected Cases in Decreasing Phase
(f) Spatially Weighted Level of Infected Cases
(g) DSE
(h) Spatially Weighted DSE
Coefficients
Waves
lag
1
2
3
4
5
6
7

(Cont'd)

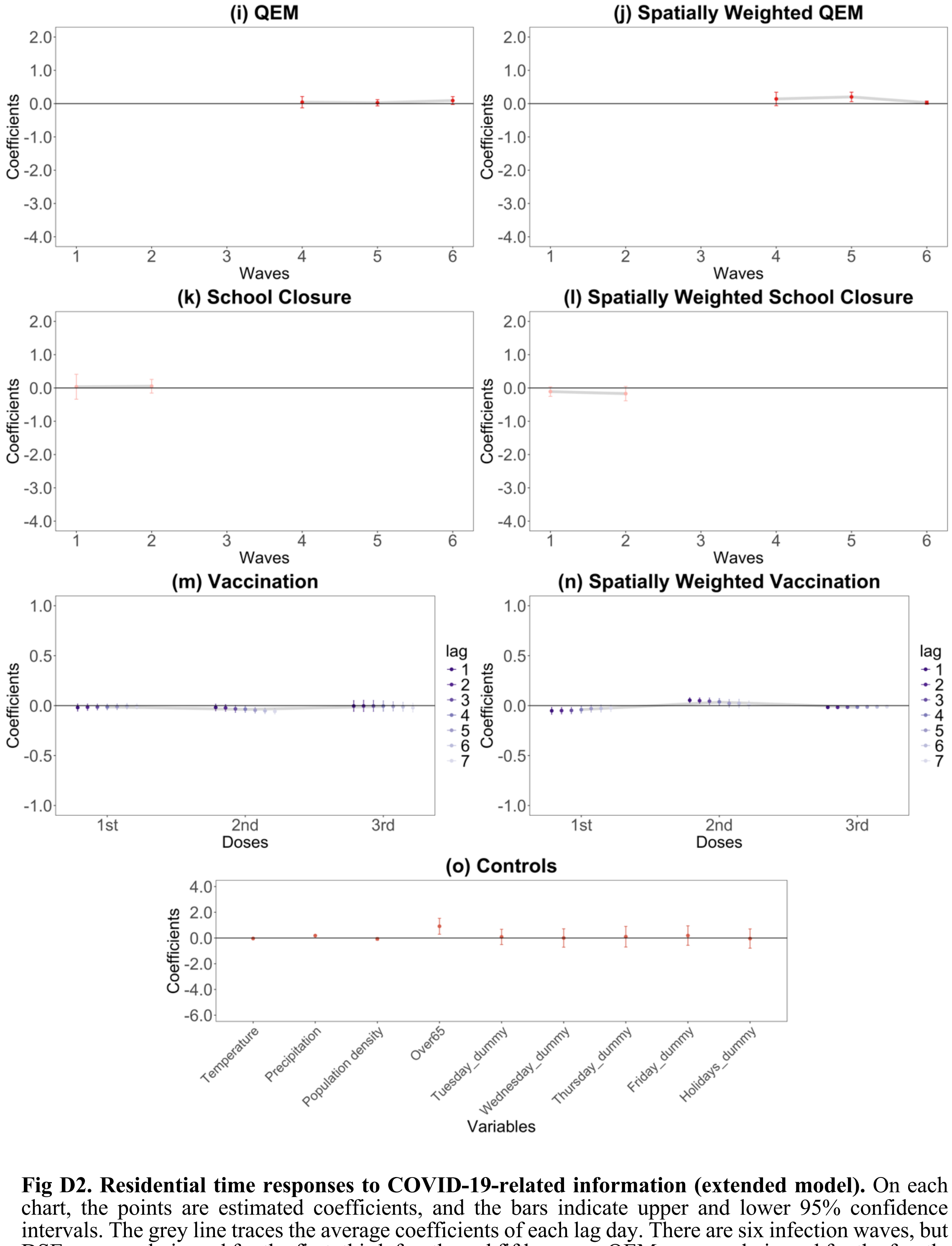

Fig D2. Residential time responses to COVID-19-related information (extended model). On each chart, the points are estimated coefficients, and the bars indicate upper and lower 95% confidence intervals. The grey line traces the average coefficients of each lag day. There are six infection waves, but DSEs were only issued for the first, third, fourth, and fifth waves; QEMs were only issued for the fourth, fifth, and sixth waves; and school closures were only requested for the first and second waves. I take a daily lag from 1 to 7 days for infected cases in the increasing phase, infected cases in the decreasing phase, spatially weighted infected cases, level of infected cases in the increasing phase, level of infected cases in the decreasing phase, spatially weighted level of infected cases, vaccination, and spatially weighted vaccination. I conduct the regression analysis seven times, from lags 1 to 7. I do not take a daily lag for the DSE, spatially weighted DSE, QEM, spatially weighted QEM, school closure, spatially weighted school closure, and control variables; these estimates are from the lag-1 regression.

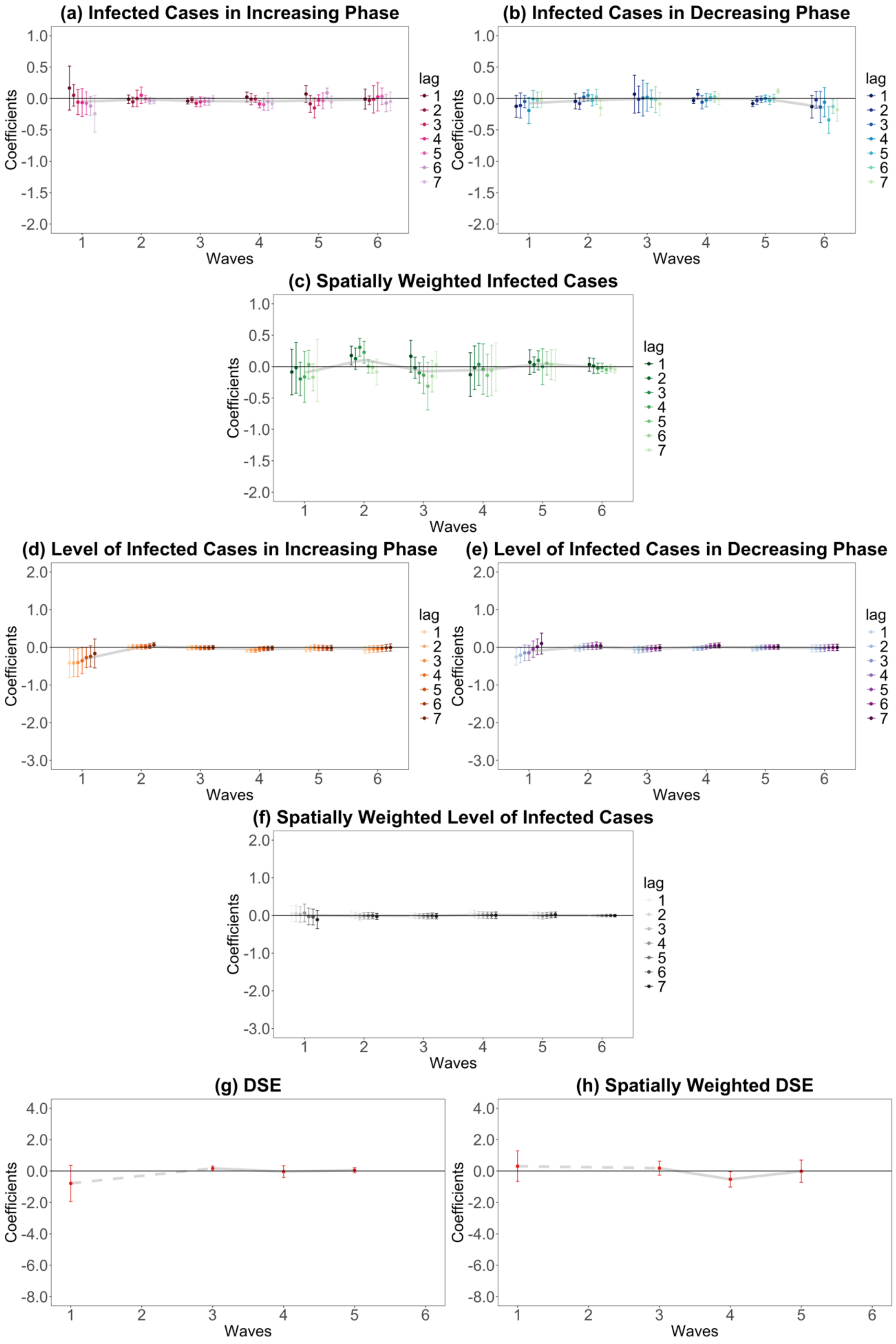
(a) Infected Cases in Increasing Phase
(b) Infected Cases in Decreasing Phase
(c) Spatially Weighted Infected Cases
(d) Level of Infected Cases in Increasing Phase
(e) Level of Infected Cases in Decreasing Phase
(f) Spatially Weighted Level of Infected Cases
(g) DSE
(h) Spatially Weighted DSE
Coefficients
Waves
lag
1
2
3
4
5
6
7
1.0
0.5
0.0
-0.5
-1.0
-1.5
-2.0
2.0
-3.0
4.0
-4.0
-6.0
-8.0

(Cont'd)

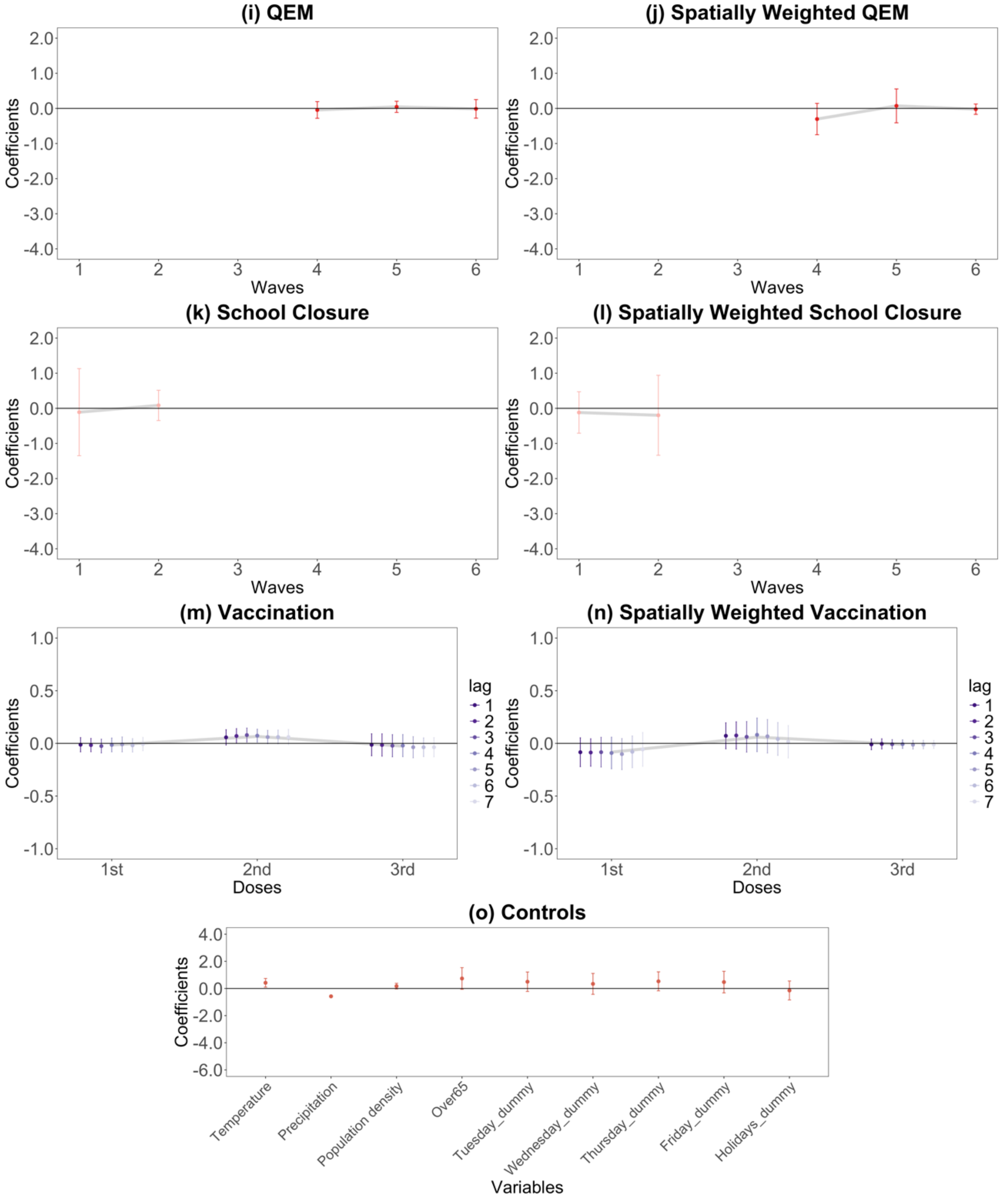

Fig D3. Grocery and pharmacy human mobility responses to COVID-19-related information (extended model). On each chart, the points are estimated coefficients, and the bars indicate upper and lower 95% confidence intervals. The grey line traces the average coefficients of each lag day. There are six infection waves, but DSEs were only issued for the first, third, fourth, and fifth waves; QEMs were only issued for the fourth, fifth, and sixth waves; and school closures were only requested for the first and second waves. I take a daily lag from 1 to 7 days for infected cases in the increasing phase, infected cases in the decreasing phase, spatially weighted infected cases, level of infected cases in the increasing phase, level of infected cases in the decreasing phase, spatially weighted level of infected cases, vaccination, and spatially weighted vaccination. I conduct the regression analysis seven times, from lags 1 to 7. I do not take a daily lag for the DSE, spatially weighted DSE, QEM, spatially weighted QEM, school closure, spatially weighted school closure, and control variables; these estimates are from the lag-1 regression.

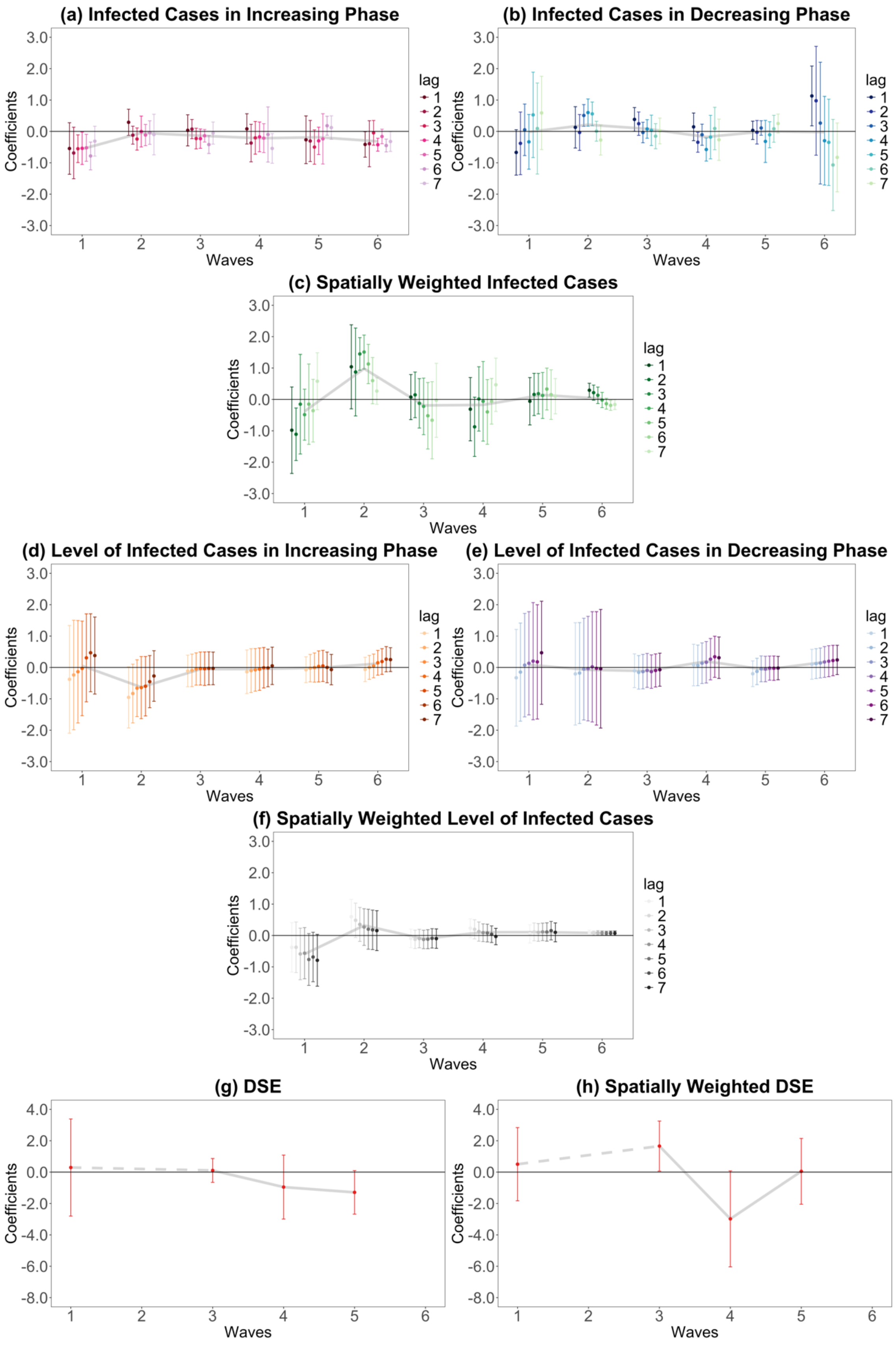
(a) Infected Cases in Increasing Phase
(b) Infected Cases in Decreasing Phase
(c) Spatially Weighted Infected Cases
(d) Level of Infected Cases in Increasing Phase
(e) Level of Infected Cases in Decreasing Phase
(f) Spatially Weighted Level of Infected Cases
(g) DSE
(h) Spatially Weighted DSE
Coefficients
Waves
lag
1
2
3
4
5
6
7
3.0
2.0
1.0
0.0
-1.0
-2.0
-3.0
4.0
-4.0
-6.0
-8.0

(Cont'd)

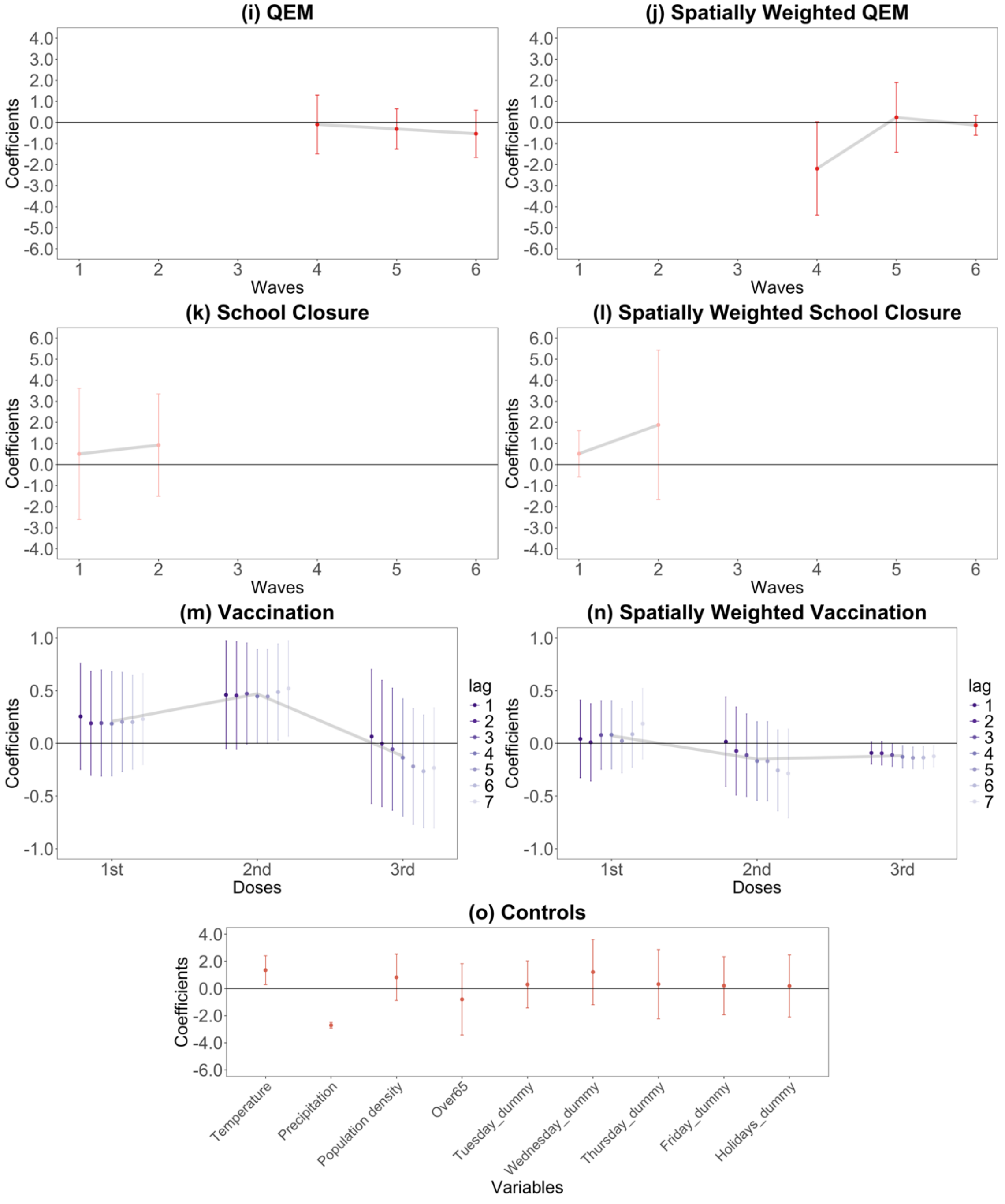

Fig D4. Parks human mobility responses to COVID-19-related information (extended model). On each chart, the points are estimated coefficients, and the bars indicate upper and lower 95% confidence intervals. The grey line traces the average coefficients of each lag day. There are six infection waves, but DSEs were only issued for the first, third, fourth, and fifth waves; QEMs were only issued for the fourth, fifth, and sixth waves; and school closures were only requested for the first and second waves. I take a daily lag from 1 to 7 days for infected cases in the increasing phase, infected cases in the decreasing phase, spatially weighted infected cases, level of infected cases in the increasing phase, level of infected cases in the decreasing phase, spatially weighted level of infected cases, vaccination, and spatially weighted vaccination. I conduct the regression analysis seven times, from lags 1 to 7. I do not take a daily lag for the DSE, spatially weighted DSE, QEM, spatially weighted QEM, school closure, spatially weighted school closure, and control variables; these estimates are from the lag-1 regression. Only the parks human mobility responses has a wider CI, so the vertical axis scale is different compared to the other figures.

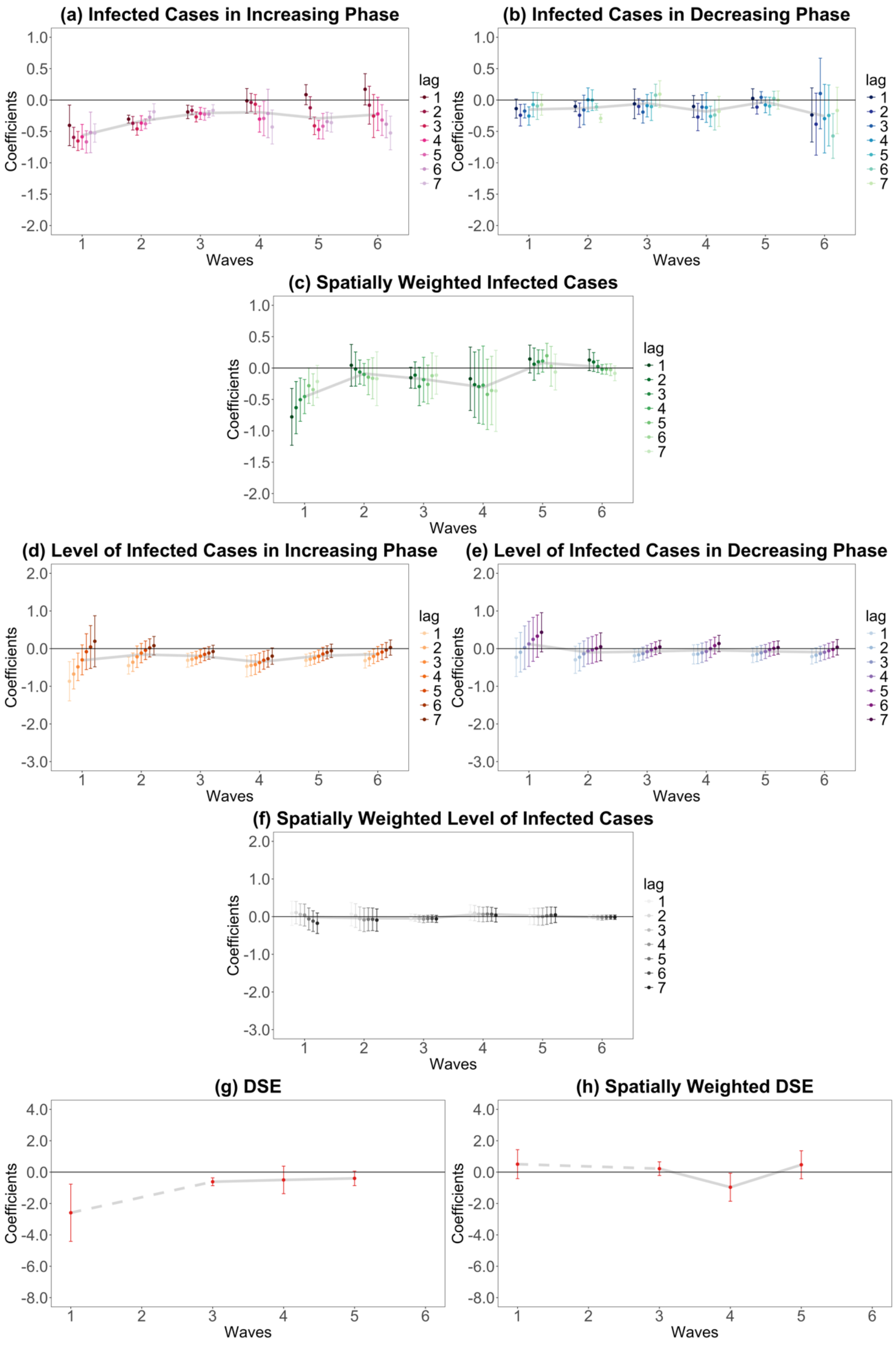
(a) Infected Cases in Increasing Phase
(b) Infected Cases in Decreasing Phase
(c) Spatially Weighted Infected Cases
(d) Level of Infected Cases in Increasing Phase
(e) Level of Infected Cases in Decreasing Phase
(f) Spatially Weighted Level of Infected Cases
(g) DSE
(h) Spatially Weighted DSE
Coefficients
Waves
lag
1
2
3
4
5
6
7

(Cont'd)

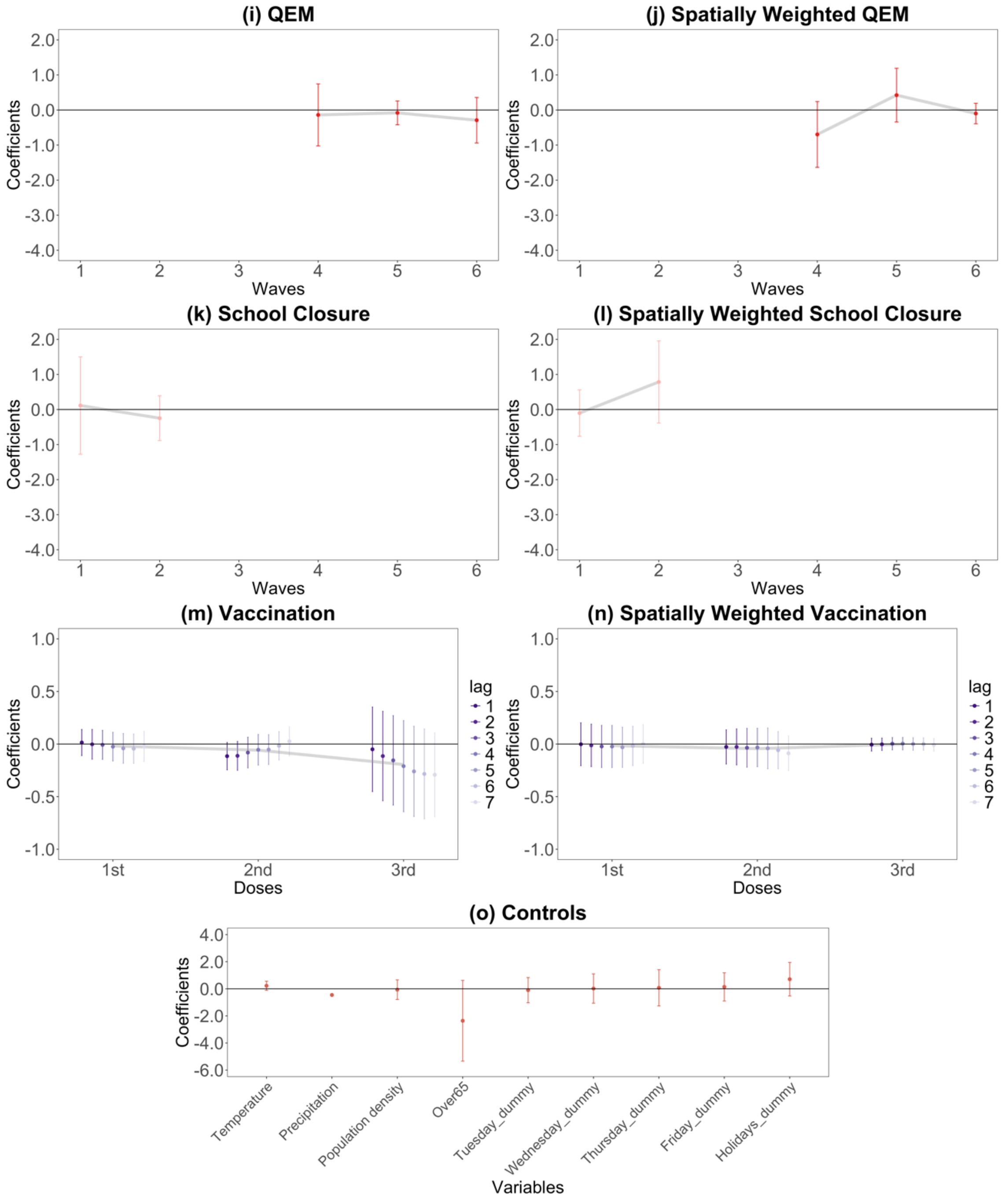

Fig D5. Transit stations human mobility responses to COVID-19-related information (extended model). On each chart, the points are estimated coefficients, and the bars indicate upper and lower 95% confidence intervals. The grey line traces the average coefficients of each lag day. There are six infection waves, but DSEs were only issued for the first, third, fourth, and fifth waves; QEMs were only issued for the fourth, fifth, and sixth waves; and school closures were only requested for the first and second waves. I take a daily lag from 1 to 7 days for infected cases in the increasing phase, infected cases in the decreasing phase, spatially weighted infected cases, level of infected cases in the increasing phase, level of infected cases in the decreasing phase, spatially weighted level of infected cases, vaccination, and spatially weighted vaccination. I conduct the regression analysis seven times, from lags 1 to 7. I do not take a daily lag for the DSE, spatially weighted DSE, QEM, spatially weighted QEM, school closure, spatially weighted school closure, and control variables; these estimates are from the lag-1 regression.

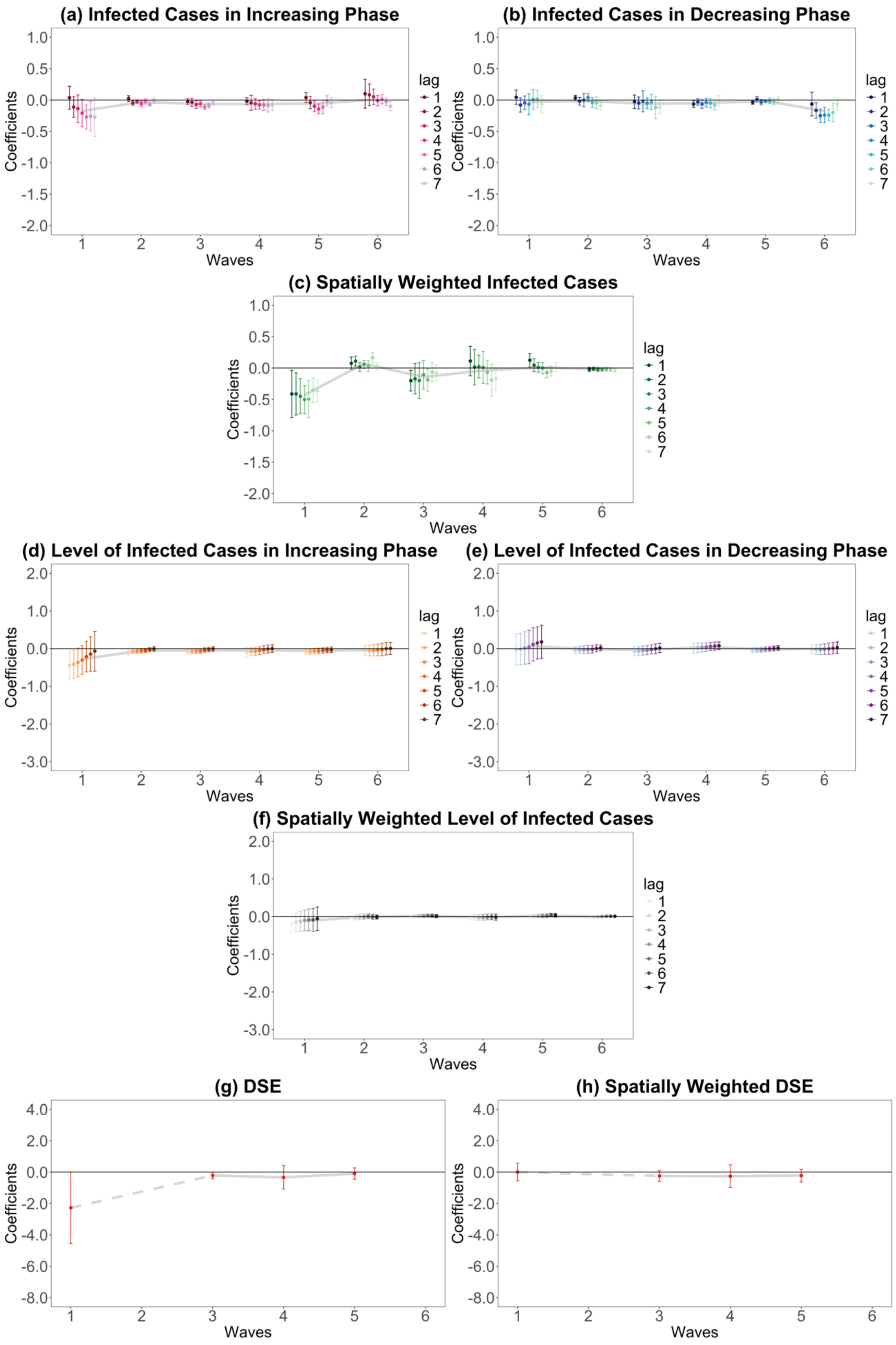
(a) Infected Cases in Increasing Phase
(b) Infected Cases in Decreasing Phase
(c) Spatially Weighted Infected Cases
(d) Level of Infected Cases in Increasing Phase
(e) Level of Infected Cases in Decreasing Phase
(f) Spatially Weighted Level of Infected Cases
(g) DSE
(h) Spatially Weighted DSE
Coefficients
Waves
lag
1
2
3
4
5
6
7

(Cont'd)

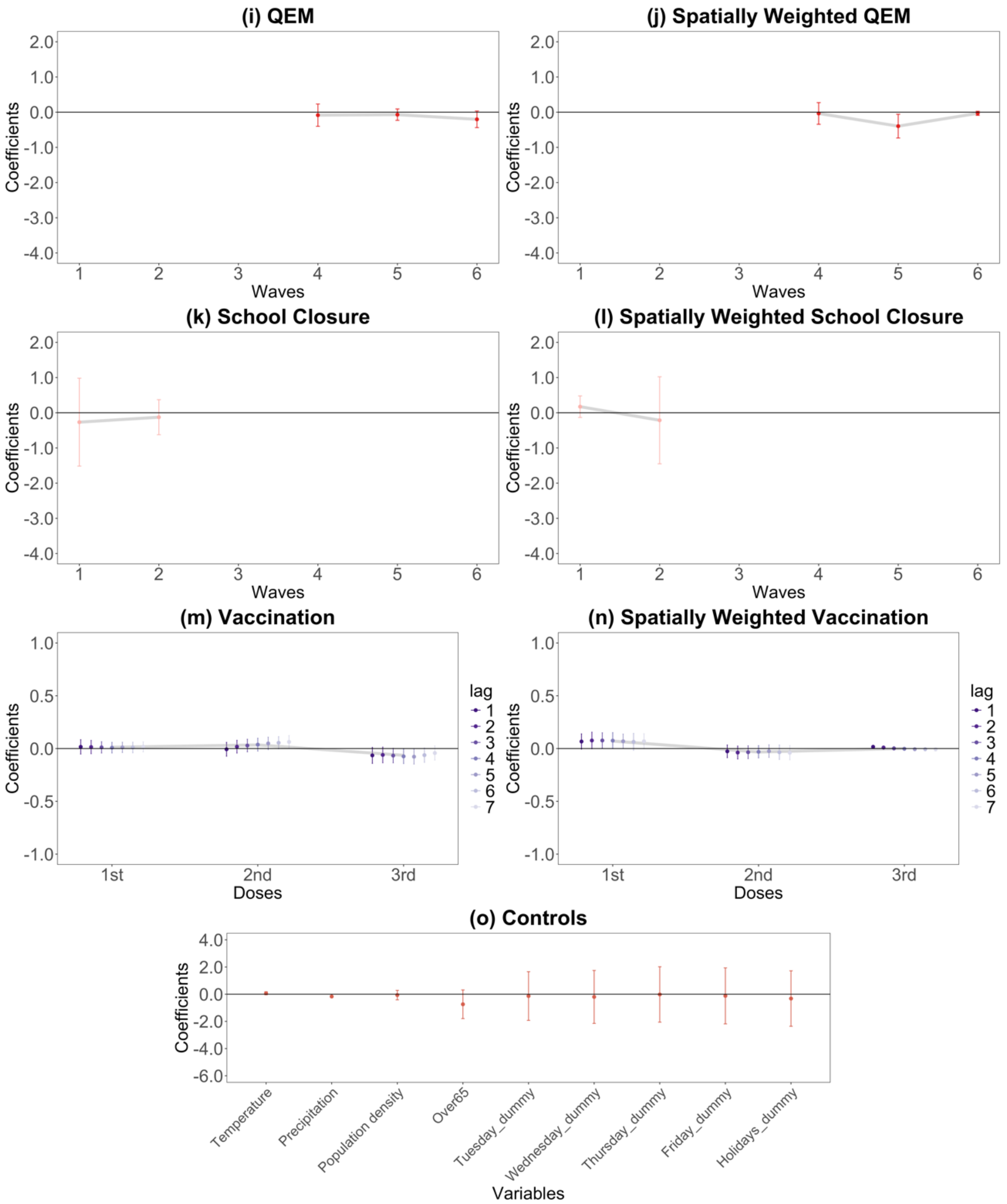

Fig D6. Workplaces human mobility responses to COVID-19-related information (extended model). On each chart, the points are estimated coefficients, and the bars indicate upper and lower 95% confidence intervals. The grey line traces the average coefficients of each lag day. There are six infection waves, but DSEs were only issued for the first, third, fourth, and fifth waves; QEMs were only issued for the fourth, fifth, and sixth waves; and school closures were only requested for the first and second waves. I take a daily lag from 1 to 7 days for infected cases in the increasing phase, infected cases in the decreasing phase, spatially weighted infected cases, level of infected cases in the increasing phase, level of infected cases in the decreasing phase, spatially weighted level of infected cases, vaccination, and spatially weighted vaccination. I conduct the regression analysis seven times, from lags 1 to 7. I do not take a daily lag for the DSE, spatially weighted DSE, QEM, spatially weighted QEM, school closure, spatially weighted school closure, and control variables; these estimates are from the lag-1 regression.

References

1. Lawrence R. The coronavirus and Japan's Constitution. The Japan Times. 2020 April 14 [cited 2022 October 28]. Available from: https://www.japantimes.co.jp/opinion/2020/04/14/commentary/japan-commentary/coronavirus-japans-constitution/?appsule=32

2. Watanabe T, Yabu T. Japan's voluntary lockdown. PLoS One. 2021;16:e0252468. https://doi.org/10.1371/journal.pone.0252468

3. Watanabe T, Yabu T. Japan's voluntary lockdown: Further evidence based on age-specific mobile location data. Jpn Econ Rev. 2021;72:333–370. https://doi.org/10.1007/s42973-021-00077-9

4. Yabe T, Tsubouchi K, Fujiwara N, Wada T, Sekimoto Y, Ukkusuri SV. Non-compulsory measures sufficiently reduced human mobility in Tokyo during the COVID-19 epidemic. Sci Rep. 2020;10:18053. https://doi.org/10.1038/s41598-020-75033-5

5. Tokyo Metropolitan Government. Information on Tokyo Emergency Measures, etc. 2022 [cited 2022 October 28]. Available from: https://www.bousai.metro.tokyo.lg.jp/1007617/index.html

6. Osaka Prefectural Government. Efforts to prevent the spread of infection (Requests in the past, etc.). 2022 [cited 2022 October 28]. Available from: https://www.pref.osaka.lg.jp/kikaku/kinkyuzitai-yousei/index.html

7. Novel Coronavirus Response Headquarters, Prime Minister's Office of Japan. The Basic Action Policy. 2023 [cited 2024 March 26]. Available from: https://www.kantei.go.jp/jp/singi/novel_coronavirus/taisaku_honbu.html

8. Novel Coronavirus Response Headquarters, Prime Minister's Office of Japan. Report on the Implementation Status of the declaration of a state of emergency of COVID-19. 2021 October [cited 2024 March 26]. Available from: https://www.kantei.go.jp/jp/singi/novel_coronavirus/th_siryou/houkoku_r031008.pdf

9. Japan Broadcasting Corporation (NHK). Government decides on new financial support measures to accelerate vaccination (in Japanese). 2021 May 25 [cited 2022 October 28]. Available from: https://www3.nhk.or.jp/news/html/20210525/k10013049871000.html

10. Ministry of Health, Labour and Welfare. Vaccines scheduled to be supplied in Japan (those to be supplied starting this year (2021) and next year (2022)) (in Japanese). 2021 [cited 2024 March 19]. Available from: https://www.cov19-vaccine.mhlw.go.jp/qa/uploads/5-1.pdf

11. Ministry of Health, Labour and Welfare. COVID-19 Q&A (in Japanese). 2022 [Cited 2022 August 15]. Available from: https://www.mhlw.go.jp/stf/seisakunitsuite/bunya/vaccine_qa.html

12. Google. COVID-19 community mobility reports; 2022 [cited 2022 August 16]. Available from: https://www.google.com/covid19/mobility/

13. Japan Broadcasting Corporation (NHK). Number of cases of infection by prefecture. special site: COVID-19 (in Japanese); 2022 [cited 2022 August 16]. (Currently available from the Ministry of Health, Labour and Welfare. The data shows - Information on COVID-19 infections - (in Japanese); 2024 [cited 2024 July 5]. https://covid19.mhlw.go.jp)

14. Cabinet Secretariat. The state of emergency (in Japanese); 2020 [cited 2022 October 28]. (Currently available from the Cabinet Agency for Infectious Disease Crisis Management. About COVID-19 – Others (in Japanese); 2024 [cited 2024 July 5]. https://www.caicm.go.jp/information/citizen/corona/index.html)

15. Digital Agency. Open data on the vaccination status of COVID-19 vaccines (in Japanese); 2022 [cited 2022 August 16]. (Currently available from the Ministry of Health, Labour and Welfare. The number of COVID-19 vaccines administered, open data (in Japanese); 2024 [cited 2024 July 5]. https://www.mhlw.go.jp/stf/seisakunitsuite/bunya/kenkou_iryou/kenkou/kekkaku-kansenshou/yobou-sesshu/syukeihou_00002.html)

16. Damodar NG, Dawn CP. Basic Econometrics 5th edition. McGraw-Hill; 2009.
